# Spectrophotometric Studies of a Fluorescent Naphthalenediimide PET and ICT pH Indicator and DNA Intercalator

**DOI:** 10.3390/molecules31183211

**Published:** 2026-09-11

**Authors:** Alex D. Johnson, Mario Valentino, Gary J. Hunter, David C. Magri

**Affiliations:** 1 Department of Chemistry, Faculty of Science, University of Malta, MSD 2080 Msida, Malta; 2 Department of Physiology and Biochemistry, Faculty of Medicine & Surgery, University of Malta, MSD 2080 Msida, Malta

**Keywords:** naphthalenediimide, fluorescence, circular dichroism, MCF-7 cells, photoinduced electron transfer, internal charge transfer

## Abstract

Three naphthalenedimides (NDIs) were studied by UV-visible absorbance, fluorescence and circular dichroism spectroscopy in aqueous methanolic solution as DNA intercalators. Distinct solution colours are observed for the compounds under room lighting and upon irradiation with 365 nm UV light. The tetra-substituted dimethylaminoethylene NDI is designed as a fluorescent pH indicator with a ‘fluorophore–spacer–receptor’ construct based on a photoinduced electron transfer (PET) mechanism. This NDI derivative exhibits a significant change in the ellipticity with calf-thymus DNA. Fluorescence DNA titrations further revealed strong binding towards DNA, resulting in pronounced emission quenching. Incubation of MCF-7 cells reveals localisation of the fluorescent probe in the nucleus, specifically in the nucleoli.

## 1. Introduction

Naphthalenediimides (NDIs) are π-deficient polycyclic aromatic compounds routinely used as fluorescent cellular imaging agents and antineoplastics [1,2,3,4,5,6]. With many advantageous properties, including thermal and photochemical stability, and interesting electrochemical [7,8] and photophysical properties [9,10], they are suitable for the design of chemosensors [11,12,13,14], molecular logic gates [15,16,17], optoelectronic materials [18,19] and polymers [20,21,22]. While the emission of core-unsubstituted NDIs is weakly fluorescent [16], substitution of the heteroaromatic planar ring results in highly coloured and fluorescent compounds that emit in the red and infra-red region [23]. Typical studies of NDIs involve absorbance, fluorescence and dichroism spectroscopy for monitoring binding with biomolecules, such as DNA and proteins [24,25,26]. Furthermore, NDIs are used as fluorescent pH probes for visualising uptake and localisation within living cells [27,28]. We are interested in NDIs as a fluorophore platform for the development of intelligent molecular logic gates for fluorescent cellular imaging and oncology [29,30].

**Figure 1:**
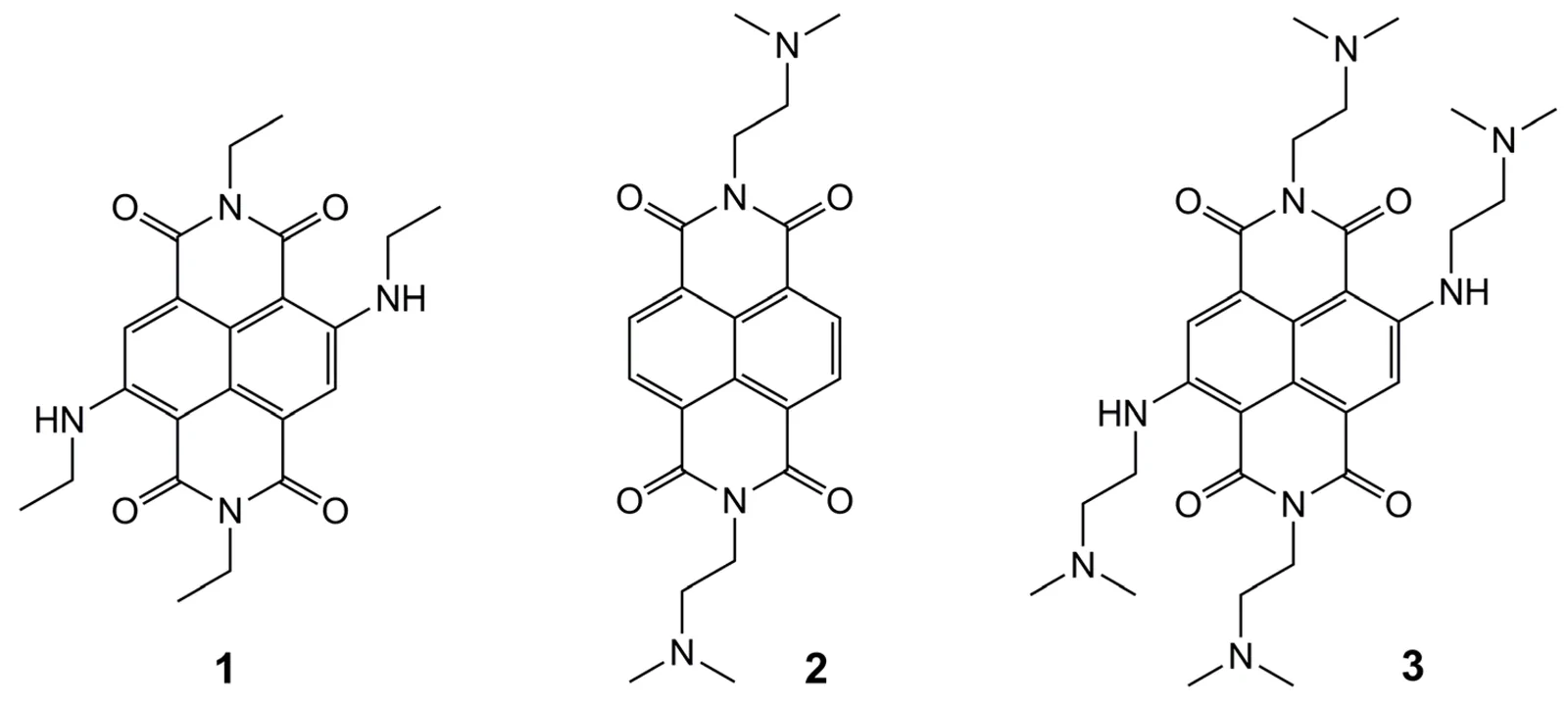
The NDIs **1**–**3** studied as fluorescent pH indicators and DNA binding agents.

Herein we report the synthesis, characterisation and photophysics of a series of NDIs **1**–**3** (Figure 1). Molecule **1** is substituted with ethyl substituents at the imide and aminoethyl at the 2,6-core positions. Molecule **2** is substituted with two *N*,*N*-dimethylaminoethylene units at the imide positions. Molecule **3** is appended with four *N*,*N*-dimethylaminoethylene substituents at the imide and core positions. Our aim was to access the absorbance and emission properties of the NDI derivatives, and the supramolecular interactions with double-stranded DNA. The photochemical properties were studied by UV-vis absorbance and steady-state fluorescence spectroscopy, including spectrophotometric pH titrations. The interaction with calf-thymus DNA (CT-DNA) was analysed by absorption and fluorometric binding studies and circular dichroism (CD). The fluorescent cellular imaging properties of molecule **3** was examined in MCF-7 human breast cancer cells.

## 2. Results and Discussion

Compounds **1** and **3** were synthesised from 2,6-dibromonaphthalene-1,4,5,8-tetracarboxylic dianhydride, and **2** was synthesised from 1,4,5,8-naphthalenetetracarboxylic dianhydride with excess amine in DMF, using microwave-assisted heating. The percent yields of compounds **1**–**3** were 60%, 96% and 42% after recrystallisation from DMF or ethyl acetate. The compounds were confirmed by ^1^H, ^13^C NMR and FTIR spectroscopy (Figures S1–S9) and studied in 1:1 (*v*/*v*) methanol/water by UV-visible absorbance, fluorescence and circular dichroism spectroscopy.

### 2.1. UV-Vis Absorbance and Fluorescence Spectroscopy

**Figure 2:**
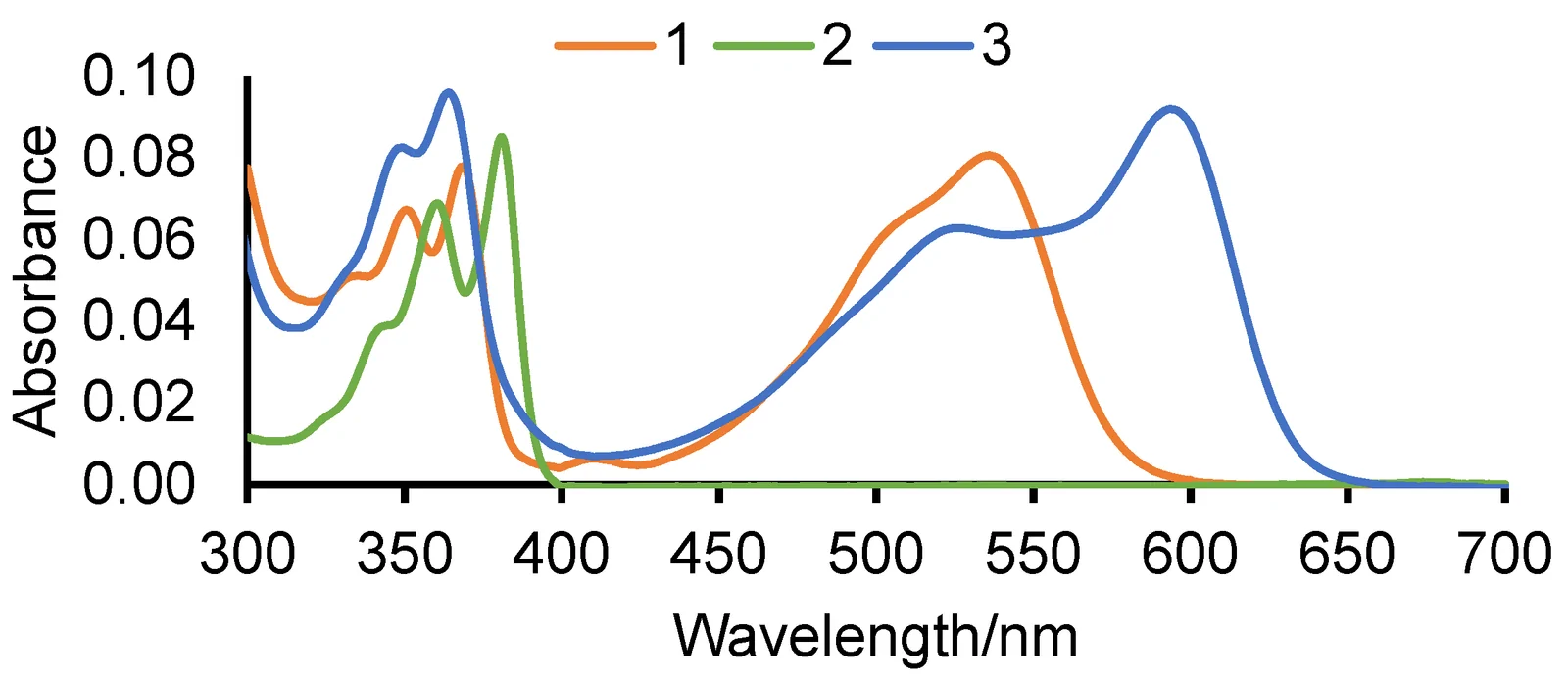
Overlaid UV-vis absorption spectra of 3 μM of compounds **1**–**3** in 1:1 (*v*/*v*) methanol/water.

**Figure 3:**
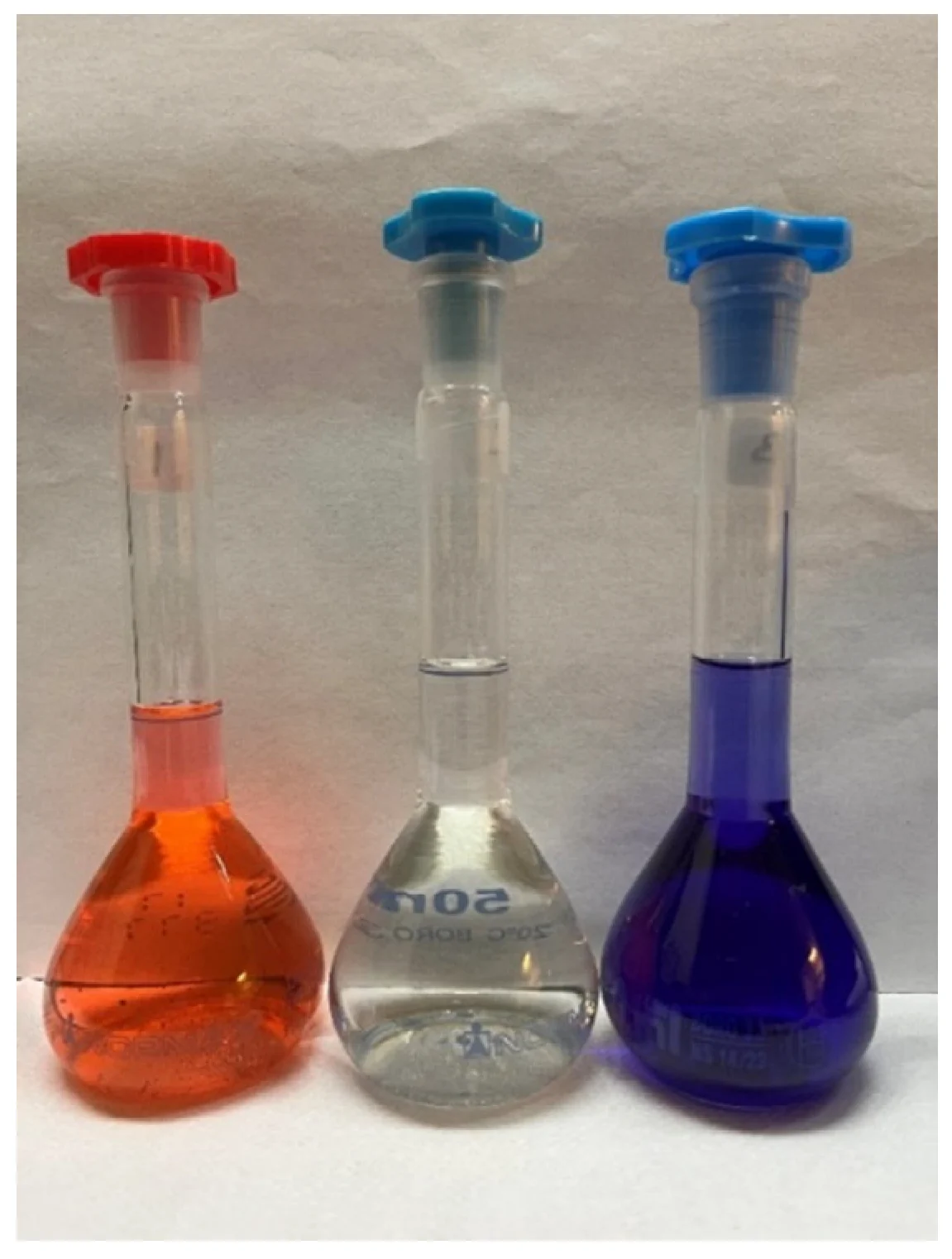
1:1 (*v*/*v*) methanol/water solutions of compounds **1**–**3** under room white lighting.

**Figure 4:**
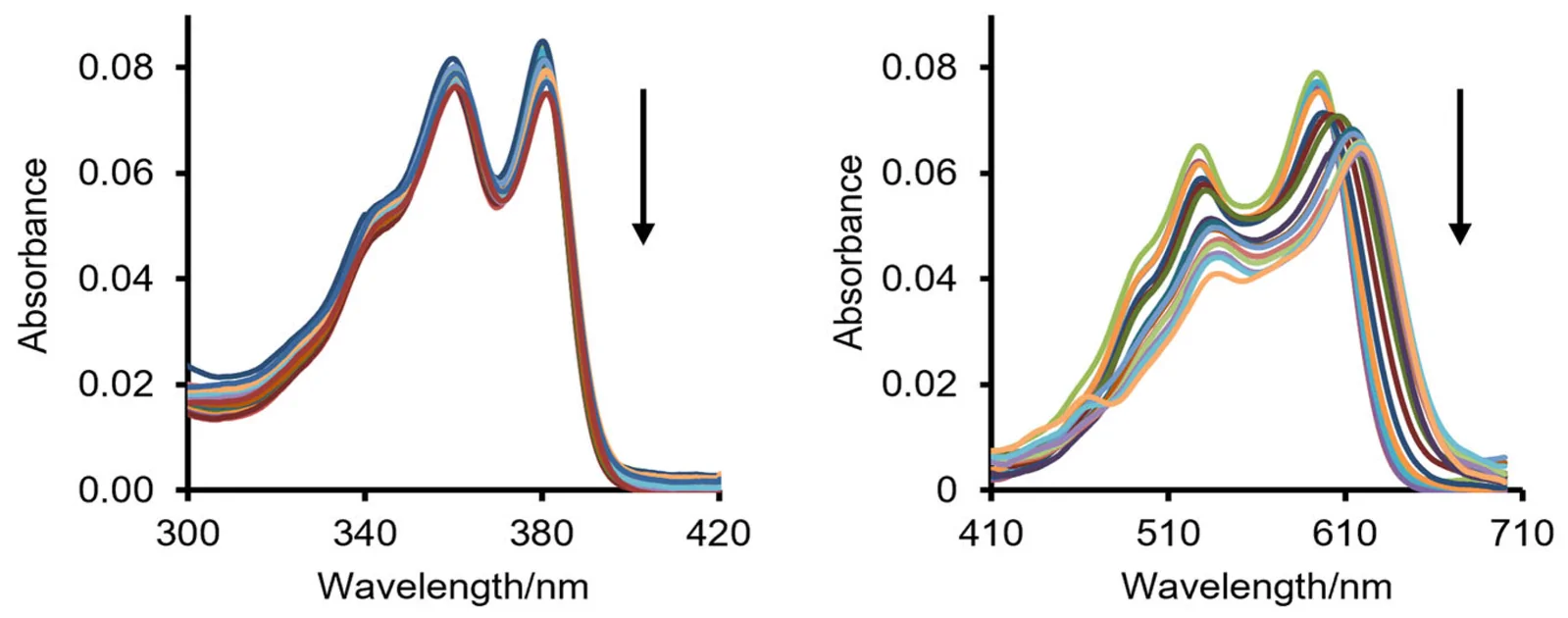
UV-vis absorbance spectra of 3 μM of compound **2** and 4 μM of compound **3** in 1:1 (*v*/*v*) methanol/water between pH 3–11. The peaks shift downwards with increasing [H^+^].

The UV-visible spectra of compounds **1**–**3** in 1:1 (*v*/*v*) methanol/water are shown in Figure 2. The contrasting solution colours of orange, colourless and blue foreshadowed different UV-vis profiles (Figure 3). The UV-vis spectra have absorption bands ranging from 200 to 400 nm for compound **2** and 200 to 700 nm for **1** and **3**. Maxima at pH 11 occur at 553 nm, 382 nm and 594 nm for compounds **1**–**3**, respectively. The corresponding molar absorption coefficients, expressed as log ε, are relatively large at 3.48, 4.21 and 4.00. These values are consistent with a π→π* electronic transition attributed to the NDI fluorophore. At pH 4, the absorbance maxima of compound **2** are slightly blue, shifted by 2 nm, while for compound **3** the peak maxima are greatly shifted on protonation to 619 nm due to the internal charge transfer (ICT) character and electric field effect on protonation. The pH-dependent UV-vis spectra of compounds **2** and **3** are shown in Figure 4. The absorbance of **1** is pH-independent between 3.0 and 11.0. The log ε of compounds **1**–**3** at pH 4.0 are 3.48, 4.41 and 4.06. No indication of precipitation or aggregation was observed at basic pH as earlier reported in aqueous media [30].

Molecule **3** is a fluorescent PET and ICT system employing a ‘receptor–spacer–fluorophore’ [31] format with four ethylenedimethylamino ‘spacer–receptor’ units about the NDI fluorophore. The four proton receptors are capable, when unprotonated, of PET from an amine to the excited NDI fluorophore, which results in a decreased fluorescence output. Compound **2** fits the PET model with two dimethylamino receptors separated from the fluorophore by an ethylene spacer. In principle, compounds **2** and **3** are multi-input H^+^-driven YES logic gates with *N*,*N*-dimethylethylenediamine units as proton receptors, separated by ethylene spacers from the NDI fluorophore. Compound **1** is a fluorescent PASS 1 logic gate devoid of tertiary amine receptors. Compound **2** fits the design of an ‘off–on’ H^+^-driven YES logic gate.

Similarly, compound **3** was synthesised as an ‘off–on’ H^+^-driven YES logic gate based on PET switching. The molecule contains four *N*,*N’*-dimethylamino proton receptors, two stemming from each imide position, and two branching from the 2- and 6-position of the naphthalene core. The amine and fluorophore are separated by ethylene spacers. Unlike compound **2**, compound **3** exhibits both PET and ICT mechanisms. The PET mechanism turns off the fluorescent intensity without any change in the emission wavelength. The ICT mechanism causes a change in the absorbance observed as a bathochromic shift in the UV-vis spectrum on going from acidic to basic pHs.

**Figure 5:**
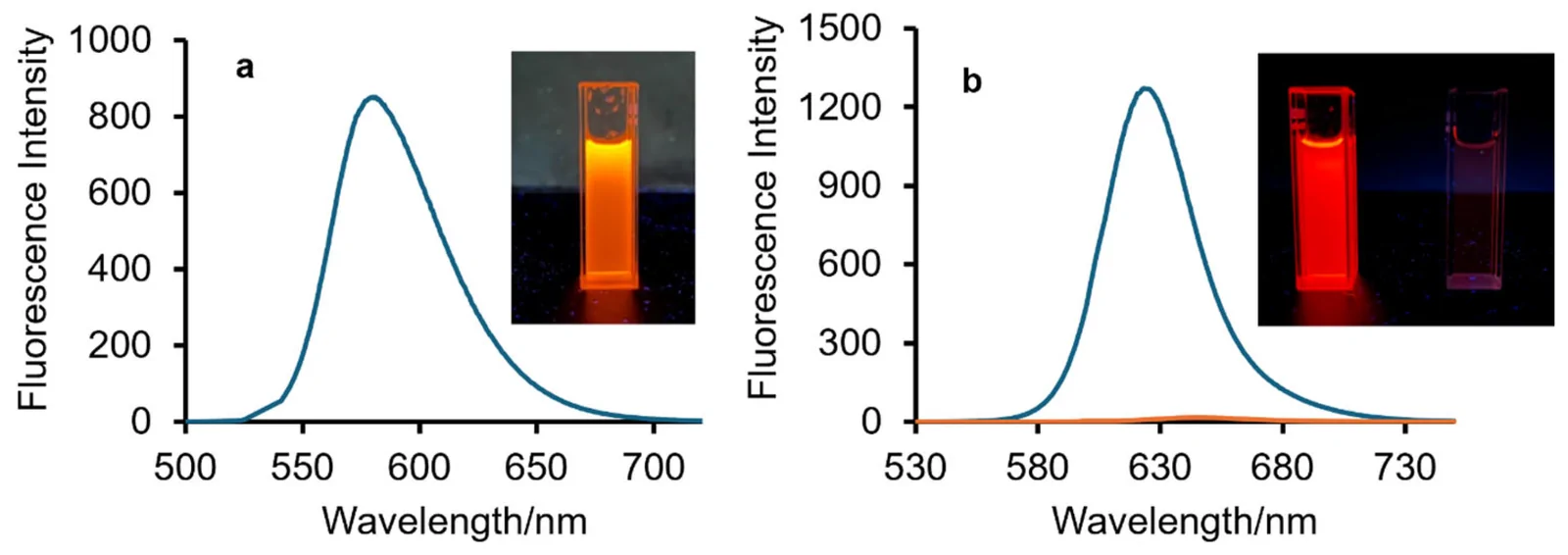
Emission spectra of (**a**) 14.7 μM of compound **1** and (**b**) 8.0 μM of compound **3** excited at 535 nm and 604 nm, respectively, in 1:1 (*v*/*v*) methanol/water in the presence of 10^−4^ M H^+^ (blue curve) and 10^−10^ M H^+^ (orange curve near baseline). The photos were taken upon irradiating with a 365 nm UV lamp. Compound **2** is non-fluorescent (Figure S12).

Compound **1** was designed as a PASS 1 logic gate, a model for comparison with compound **3**. Indeed, it gives a strong fluorescence independent of any external input (Figure 5). The fluorescence quantum yield (*Φ*_F_) is 32% in 1:1 (*v*/*v*) methanol/water. Compound **2** is a one-input H^+^-driven YES logic gate by design but functions as a PASS 0 logic gate. Such delinquency was reported for a dibutylated NDI derivative by Politi due to fast deactivation of the S1 state via intersystem crossing to a close-lying triplet state [32]. For this reason, compound **2** has no utility as an ‘off–on’ fluorescent switching device for the ‘on’ state is not visible to the naked eye (Figure S12). However, the addition of secondary nitrogen atoms on the aromatic core reconciles the fluorescence properties. The effective switching fluorescent enhancement of compound **3** is 190-fold with a *Φ*_F_ = 0.38. Moreover, the apparent p*K*_a_ of 6.0 is suited for cell imaging given its proximity to internal cytoplasm pH. The fluorescent emission colour of **1** and **3** are orange and red (Figure 5).

**Figure 6:**
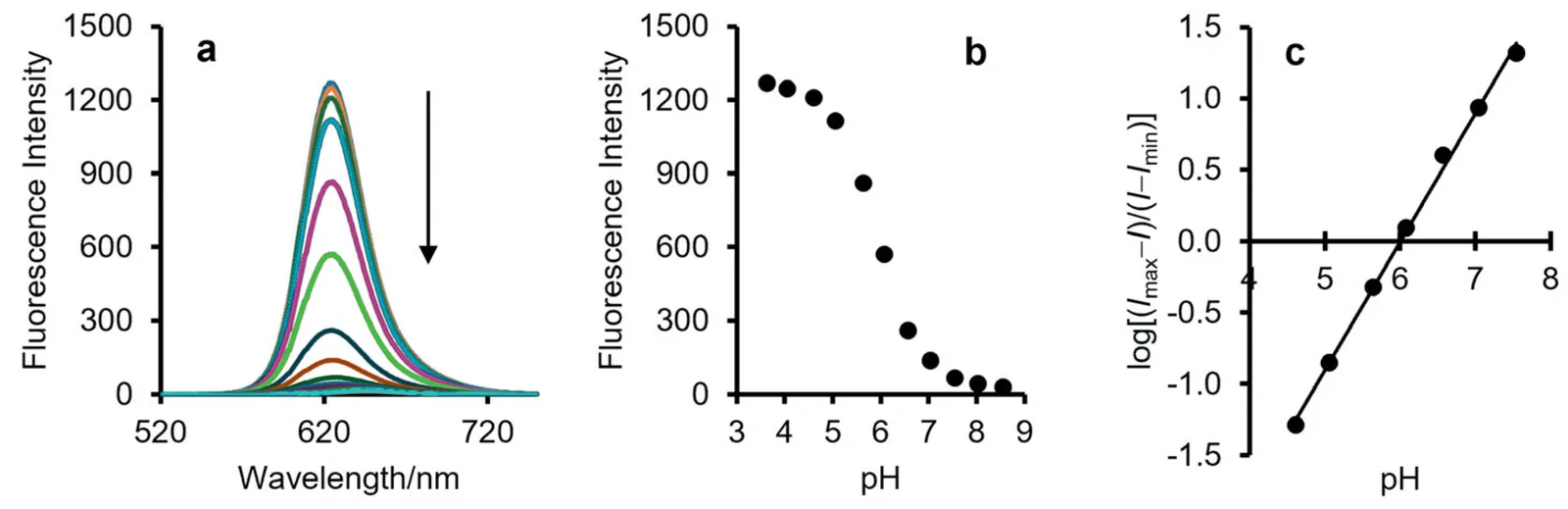
pH titration of 8 μM of compound **3** in 1:1 (*v*/*v*) methanol/water: (**a**) emission spectra with increasing addition of OH^−^, (**b**) *I*_max_ vs. pH plot, (**c**) linearised Henderson–Hasselbalch plot indicating an apparent p*K*_a_ of 6.0.

The apparent p*K*_a_ agree with simpler fluorescent switches containing the same dimethylamino receptor in methanol [33]. The proton binding constants (p*K*_a_) were determined by fluorimetric titrations according to the Henderson–Hasselbalch Equation (1):

log[(*I*_max_ − *I*)/(*I* − *I*_min_)] = −log [H^+^] + log *K*_a_ (1)

where the p*K*_a_ is the negative logarithm of the acid dissociation constant, *I*_max_ and *I*_min_ are the maximum and minimum fluorescence intensity, and *I* is the intensity at a specific wavelength. Sigmoidal-shaped curves were obtained with respect to pH switching between the plateaus within 3 log units (Figure 6). The fit of the data in 1:1 (*v*/*v*) methanol/water gave excited-state apparent p*K*_a_s of 6.9 and 6.0 for compounds **2** and **3** (Table 1). In a prior study, potentiometric titration of compound **3** yielded three identifiable p*K*_a_ values, p*K*_a1_ = 5.6, p*K*_a2_ = 7.4 and p*K*_a3_ = 7.7, such that at pH 7, compound **3** exists as a mixture of tri- and dicationic species in a 68:26 ratio [30].

The feasibility of photoinduced electron transfer can be calculated from the Weller equation, Equation (2) [34]. The PET thermodynamics for compounds **2** and **3** from the tertiary amine to the excited NDI are dependent on the oxidation potential of the receptor, *E*_ox_, the reduction potential of fluorophore *E*_red_, the singlet excited state energy of the fluorophore, *E*_s_ and the coulombic term accounting for solvent stabilisation (e^2^/εr), where *e* is the charge of the electron, *ε* is the dielectric constant and *r* is the separation distance:

Δ*G*_PET_ = *E*_ox_ − *E*_red_ − *E*_s_ − *e*^2^/ε*r* (2)

The oxidation potential of the tertiary aliphatic amine is taken as 1.15 V. Compounds **2** and **3**, though, differ in the nature of the fluorophore. The reduction potential of the core unsubstituted NDI of compound **2** is −1.11 eV [7], and the singlet energy associated with a shorter wavelength is 3.20 eV. Assuming a coulombic term contributes 0.10 V (versus SCE), the driving for PET within compound **2** is −1.1 eV. This negative result is suggestive of PET in the Marcus inverted region [35]. However, a more competitive deactivation pathway in core-unsubstituted NDIs is rapid intersystem crossing (ISC) from the S1 excited ππ* state to an upper T4 (nπ*) state [10], hence the very weak emission *Φ*_Fmax_ < 0.001 and lack of ‘off–on’ switching. Core amino-substituted compound **3** has a reduction potential of −0.89 eV and a singlet excited state energy of 2.07 eV (600 nm). The PET driving for compound **3** is mildly exothermic at −0.13 eV, which is comparable with the first anthracene-based fluorescence pH indicators [36].

### 2.2. DNA Binding Studies

**Figure 7:**
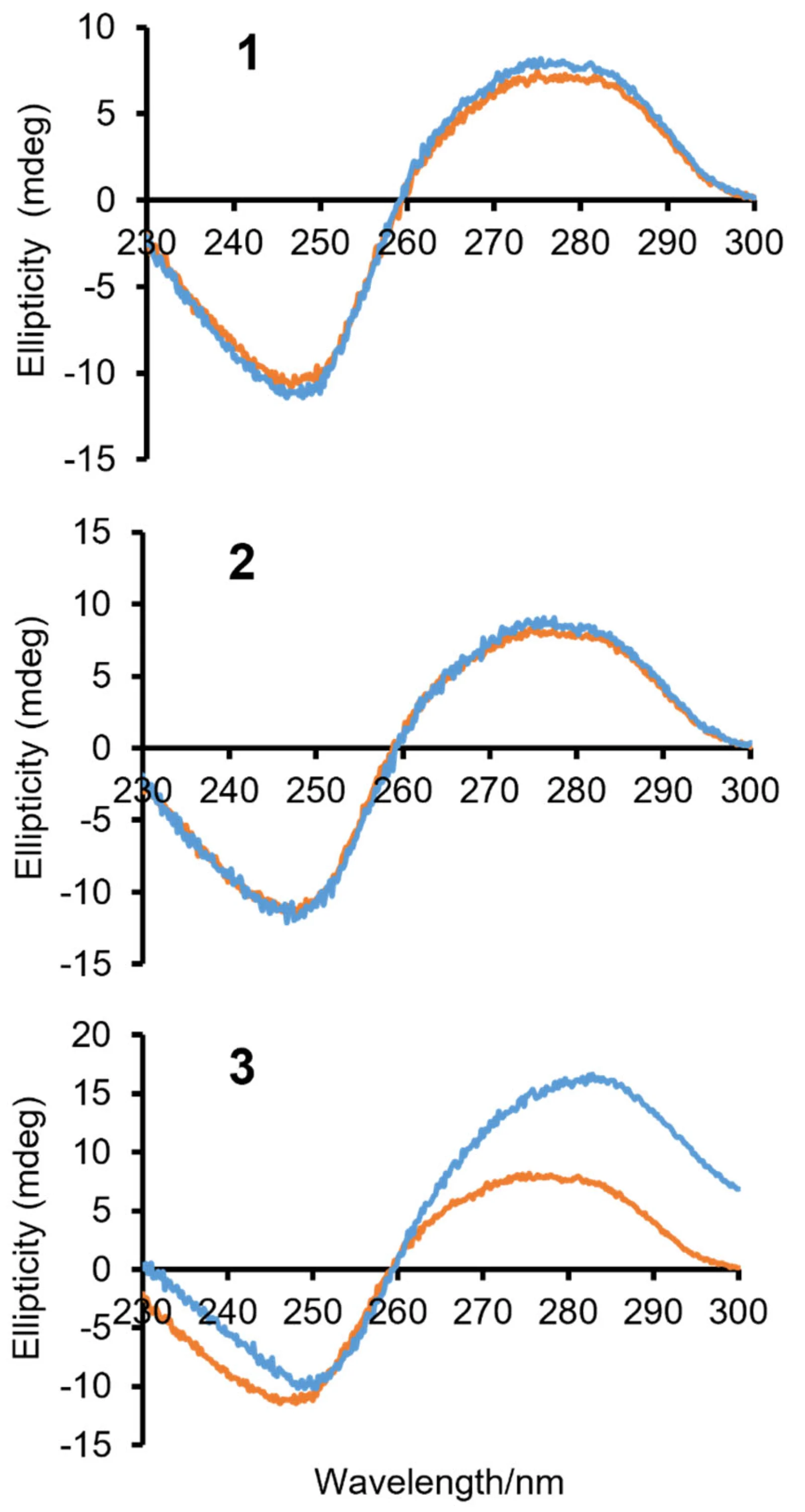
Circular dichroism spectra of 100 μM DNA (orange curve) with 20 μM of compounds **1**–**3** added (blue curve) in tris–HCl buffer solution.

Circular dichroism (CD) was used to examine the supramolecular interactions of compounds **1**–**3** with chiral double-stranded DNA, using circularly polarised electromagnetic radiation. The difference in the absorption of right- and left-handed circularly polarised light is the CD measured as the ellipticity expressed in degrees (Figure 7). The CD spectrum of CT-DNA has a helical B conformation with a characteristic negative peak at 245 nm and a positive peak at 275 nm due to base stacking [37]. CD spectra were measured in the presence of 100 μM DNA incubated with 20 μM of NDI derivatives **1**–**3**. With compounds **1** and **2**, no change was observed at 245 nm, and an ever-slight increase was observed at 275 nm, suggesting minimal change in the DNA conformation. By contrast, a significant change in the ellipticity of compound **3** was observed at 245 nm and 275 nm. Amino-substituted NDIs are known to interact with CT-DNA by π-stacking of the aromatic core in between DNA base pairs and by electrostatic interactions between the protonated positively charged amines and the negatively charged DNA phosphate backbone [38]. The change in the ellipticity of compound **3** with CT-DNA is surmised to result from a stronger binding interaction because of the four protonated dimethylamino ligands versus only two protonated ligands within compound **2**.

To further investigate the binding interaction of the NDI derivatives with CT-DNA, UV-visible absorption and fluorescence titrations were performed with compounds **1** and **3**. Due to the absence of emission from compound **2**, it was not studied further. Compound **1** displayed modest spectral changes upon incremental addition of up to 1.0 mM CT-DNA (Figure S10). The UV-vis absorption spectrum showed a gradual decrease at 537 nm and a hypochromic shift of 5 nm on increasing addition of CT-DNA in buffered solution (10 mM tris buffer, 1 mM EDTA). Fluorescence quenching was comparatively limited with compound **1** after the addition of 1.38 mM DNA, corresponding to an excess of nearly 50 equivalents of CT-DNA to the fluorescent probe. These observations suggest a weak association of compound **1** with CT-DNA in agreement with the minimal perturbation observed in the CD experiments.

**Figure 8:**
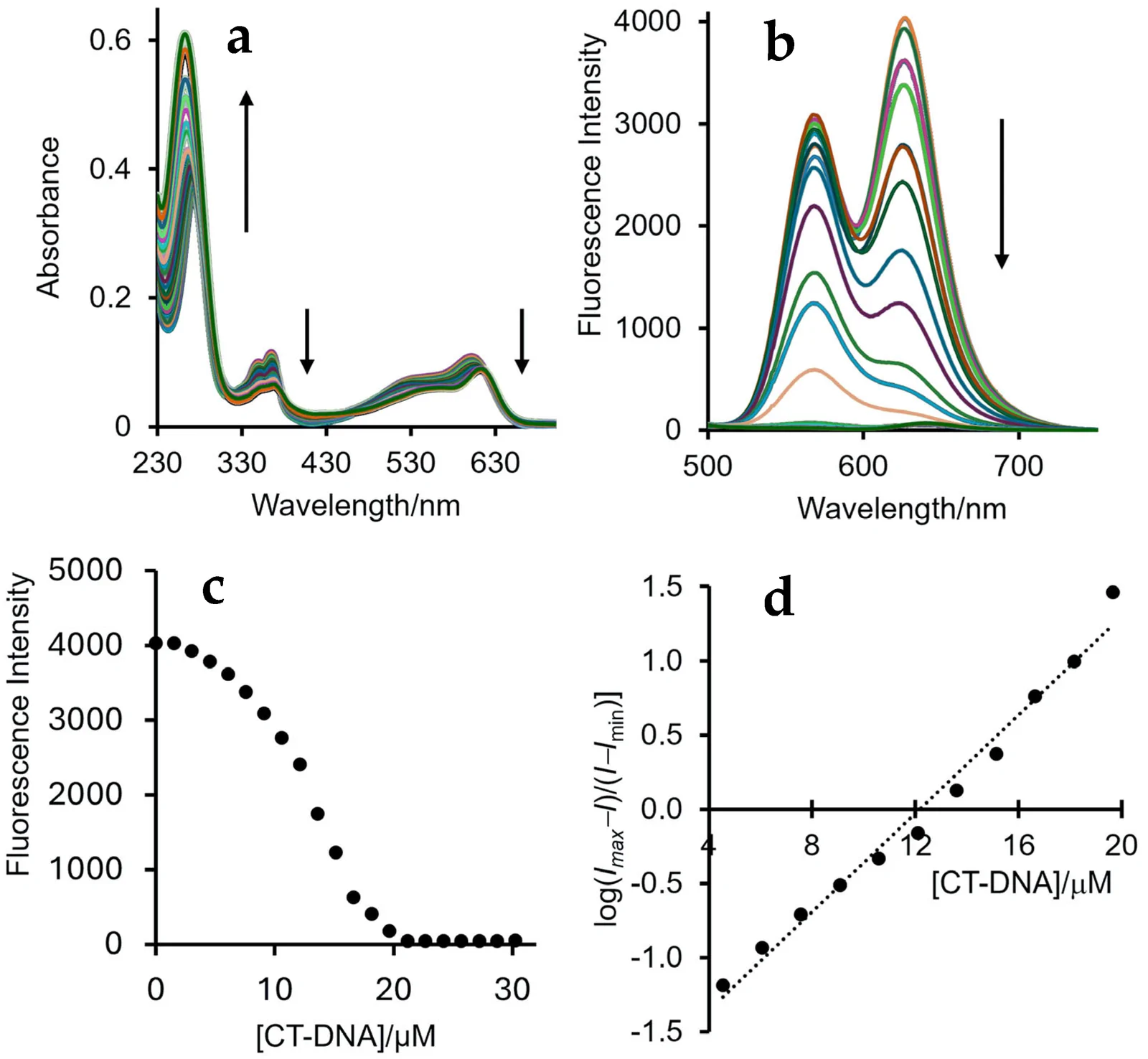
(**a**) UV-vis absorption and (**b**) emission spectra of 9.3 μM of compound **3** in 1:1 (*v*/*v*) methanol/water on addition of up to 30 μM CT-DNA (λ_ex_ = 364 nm), (**c**) emission intensity versus [CT-DNA] plot, and (**d**) linearised Henderson–Hasselbalch plot. The arrows indicate the direction of the peaks with increasing [CT-DNA].

A dramatic emission response was observed upon addition of CT-DNA to a solution of compound **3** (Figure 8). Hypochromism was observed in the visible absorption bands, and a substantial fluorescence decrease occurred at 626 nm with increasing [CT-DNA]. The addition of 12.1 μM CT-DNA resulted in a 50% quenching of compound **3**. The binding constant (*K*), taken as the reciprocal of the CT-DNA concentration from the intersection of a linearised Henderson–Hasselbalch plot (Figure 8d), is 8.3 × 10^4^ M^−1^. Analysis of the emission data by the Benesi–Hildebrand method provided *K* = 4.4 × 10^4^ M^−1^. A similar analysis of the UV-vis absorbance spectra by the Benesi–Hildebrand method gave *K* = 8.4 × 10^3^ M^−1^ (Figure S11). The magnitude of *K* indicates a strong interaction between compound **3** and CT-DNA, consistent with the significant increase in the CD spectrum. The enhanced binding affinity is attributed to the four terminal dimethylamino groups, which are partially protonated under the experimental conditions [30], facilitating favourable electrostatic interactions with the negatively charged phosphate backbone in addition to the core naphthalenediimide π-stacking capability between nucleotide base pairs [38]. Collectively, the UV-visible, fluorescence and CD studies support a strong supramolecular interaction between compound **3** and CT-DNA.

### 2.3. Fluorescent Cellular Imaging

**Figure 9:**
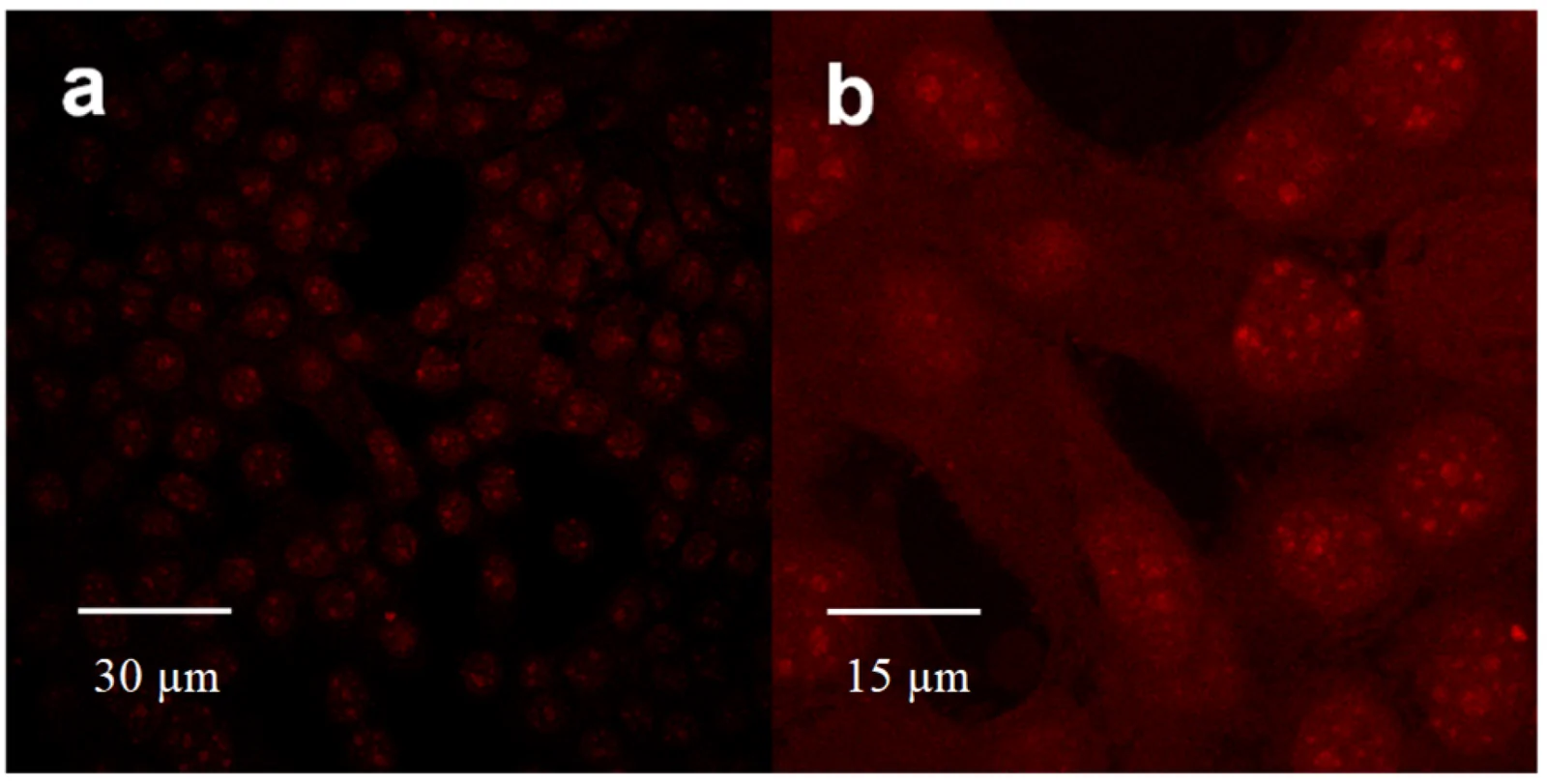
Fluorescent cellular images of MCF-7 cells incubated with 80 μM of compound **3** at 40× (**a**) and 160× (**b**) magnification after excitation with a 488 nm laser highlighting the nucleoli.

In Figure 9 fluorescent cellular images of MCF-7 cells incubated with compound **3** were viewed by confocal fluorescence microscopy. The compound primarily localises in the nucleus, specifically in the nucleoli, with some widespread distribution after 30 min exposure at 80 μM [29]. These observations are consistent with compound **3** permeating the cell membrane, and after accumulation within the nucleus, the enhanced fluorescence observed in the nucleoli is suggestive of DNA binding. In the unprotonated form, compound **3** is hydrophobic with an octanol–water partition coefficient (Log P) of 2.90, while in acidic solution it is soluble with a Log P = −1.316. The cytotoxicity of compound **3** in MCF-7 cells is reportedly 9.2 nM [29].

## 3. Materials and Methods

### 3.1. Materials

2,6-Dibromonaphthalene-1,4,5,8-tetracarboxylic dianhydride (≥98%, TCI, Tokyo, Japan), 1,4,5,8-naphthalenetetracarboxylic dianhydride (≥98%, Sigma-Aldrich, St. Louis, MO, USA), *N*,*N*-dimethylethylenediamine (≥98.0%, Sigma-Aldrich), calf-thymus DNA (10 mg/mL, Life Technologies, Carlsbad, CA, USA), triethylamine (HPLC Grade, Fisher Chemicals, Pittsburgh, PA, USA), ethylamine, DMF, dichloromethane, diethyl ether, ethyl acetate and methanol were used as received.

### 3.2. Instrumentation

Melting points were recorded at a ramp rate of 1 °C min^−1^ using a Stuart SMP40 melting point apparatus (Bibby Scientific, Stone, UK). Syntheses were performed in a 700 W SilverCrest microwave (Neckarsulm, Germany). ^1^H and ^13^C NMR spectra were obtained at a frequency of 500.13 MHz and 125.76 MHz, respectively, on a Bruker Avance III HD NMR spectrometer (Billerica, MA, USA). The chemical shifts are reported downfield from tetramethylsilane (TMS) at δ = 0.00 ppm and δ = 77.00 ppm, respectively. The acquired data was analysed using Topspin, version 4.0.7. FTIR spectra were recorded with a Shimadzu IR-Affinity-1 spectrophotometer (Kyoto, Japan) calibrated against 1601 cm^−1^ polystyrene absorption peak as a thin film on NaCl plates. UV-visible absorption spectra were obtained on a Jasco V-650 spectrophotometer (Hachioji, Japan). Fluorescence spectra were obtained on a Jasco FP-8300 spectrofluorometer. Experiments were performed at room temperature in 10 × 10 × 30 mm Suprasil quartz cuvettes.

### 3.3. Chemical Synthesis

2,6-Dibromonaphthalene-1,4,5,8-tetracarboxylic dianhydride (200 mg, 0.47 mmol) was reacted with 4 mL of excess ethylamine in DMF and 1 mL of triethylamine using a microwave for 20 min at low power (400 W) at 30 s pulse intervals. Product **1** was recrystallised from DMF and washed with diethyl ether to give 116 mg of red powder. Yield 60%; m.p. 256–259 °C; ^1^H NMR (500 MHz, CDCl_3_): *δ* 10.03 (br s, 2H, NH), 8.27 (s, 2H, Ar-H), 4.25 (q, 4H, *J* = 7.8 Hz, -CH_2_), 3.64 (q, 4H, *J* = 7.8 Hz, -CH_2_), 1.49 (t, 6H, *J* = 7.8 Hz, -CH_3_); 1.35 (t, 6H, *J* = 7.8 Hz, -CH_3_); ^13^C NMR (126 MHz, CDCl_3_): *δ* 161.82, 151.71, 138.28, 127.53, 123.57, 36.50, 35.58, 14.74, 13.19; IR *v*_max_ (NaCl/cm^−1^): 3034, 2982, 2919, 2819, 1707, 1680, 1638, 1585, 1521, 1450, 1364, 1265, 1240, 789, 750.

1,4,5,8- naphthalenetetracarboxylic dianhydride (200 mg, 0.75 mmol) was reacted with 2 mL of excess *N*, *N*-dimethylethylenediamine in DMF using a microwave for 20 min at low power (400 W) at 30 s pulse intervals. The product was precipitated from solution, filtered, and washed with diethyl ether. Product **2** was recrystallised from DMF and washed with diethyl ether to give 232 mg of yellow flakes. Yield of 96%; m.p. 291 °C (dec.); ^1^H NMR (500 MHz, CDCl_3_): *δ* 8.76 (s, 4H, Ar-H), 4.29 (t, 4H, J = 6.8 Hz, -CH_2_), 2.55 (t, 4H, J = 6.8 Hz, -CH_2_), 2.34 (s, 12H, -CH_3_); ^13^C NMR (126 MHz, CDCl_3_): *δ* 162.93, 130.99, 126.65, 56.97, 45.80, 38.68; IR *v*_max_ (NaCl/cm^−1^): 3078, 2978, 2947, 2819, 2760, 1701, 1654, 1581, 1452, 1352, 1244, 1159, 1043, 770.

Compound **3** has been reported [29,30]. 2,6-Dibromonaphthalene-1,4,5,8-tetracarboxylic dianhydride (100 mg, 0.23 mmol) was reacted with 4 mL of excess *N*,*N*-dimethylethylenediamine in DMF in the presence of Cs_2_CO_3_ (200 mg) using a microwave for 20 min at low power (400 W) at 30 s pulse intervals. Product **3** was extracted with dichloromethane, recrystallised from ethyl acetate and washed with diethyl ether to give 57 mg of purple powder. Yield 42%; m.p. 301 °C (dec); ^1^H NMR (500 MHz, CDCl_3_): *δ* 10.17 (2H, NH), 8.13 (s, 2H, Ar-H), 4.33 (m, 4H, -CH_2_), 3.58 (m, 4H, -CH_2_), 2.71 (m, 4H, -CH_2_), 2.65 (m, 4H, -CH_2_), 2.37 (s, 24H, -CH_3_); ^13^C NMR (126 MHz, CDCl_3_): *δ* 163.20, 149.03, 125.77, 125.77, 118.46, 102.08, 58.19, 56.97, 45.77, 45.59, 41.07, 38.10; IR *v*_max_ (NaCl/cm^−1^): 3292, 2972, 2943, 2818, 2766, 1680, 1636, 1578, 1447, 1458, 1314, 1184, 1042, 787.

### 3.4. UV-Vis Absorbance and Fluorescence Measurements

Solutions of compounds **1**–**3** were prepared in 250 mL volumetric flasks in 1:1 (*v*/*v*) methanol/water and diluted to an absorbance of 0.1. The pH was adjusted with 0.10 M or 0.010 M aqueous solutions of NaOH and HCl. The molar absorption coefficients *ε* were determined from the Beer–Lambert Law Equation (3), where *A* is the absorbance, *c* is the concentration, and *l* is the cuvette path length, 1.0 cm. Emission spectra were obtained by excitation at the UV-vis absorbance isosbestic point, where applicable. The fluorescence quantum yields (*Φ*_F_) were determined by Equation (4) from the area under the fluorescence spectra (*I*), the absorbance at the excitation wavelength (*A*) and the solvent refractive index (*η*). Fluorescein in ethanol (*Φ*_ref_ = 0.79) was used as the fluorescence quantum yield standard [39].

*A* = *ε l c* (3)

(4) $$\Phi_{F}=\Phi_{ref}\left(\frac{I}{I_{ref}}\right)\left(\frac{A_{ref}}{A}\right)\left(\frac{\eta^{2}}{\eta_{ref}^{2}}\right)$$

### 3.5. Circular Dichroism Spectroscopy of DNA

The CD spectra were recorded using a PiStar-180 spectrometer (Applied Photophysic, Charlotte, NC, USA) with a xenon lamp under a nitrogen atmosphere. Measurements were performed with a bandwidth of 3.0 nm and a sampling time per point of 0.5 s between wavelengths of 230–300 nm at 21 °C. Each sample was measured five times. The final plots are an average curve.

### 3.6. DNA Binding Studies

All solutions were prepared using distilled water. Tris-hydroxymethyl-aminomethane (Tris)/hydrochloric acid (HCl) was used at pH 7.4. The DNA stock solution was prepared by dissolving calf-thymus DNA in 50 mM tris–HCl buffer (pH 7.4) stored at 4 °C. The purity of the CT-DNA was assessed by the UV absorbance ratio (A260/A280 > 1.8), indicating minimal protein contamination. A nucleotide concentration of 2.6 × 10^−3^ M was determined using a molar absorption coefficient of ε = 6600 M^−1^ cm^−1^ at 260 nm. The concentrations of compounds **1** and **3** were 30 μM and 9 μM. Binding constants were determined from a linear fit using the Henderson–Hasselbalch and Benesi–Hildebrand methods. The stability of CT-DNA in 1:1 (*v*/*v*) methanol/water and water was monitored over several days by recording the UV-vis absorption spectra. No appreciable changes were observed confirming that the DNA remained stable throughout the experimental timeframe.

### 3.7. Confocal Fluorescence Microscopy

Confocal fluorescence microscopy was performed using an Olympus FV1000MPE laser-scanning microscope (Hachioji, Japan) equipped for confocal and multiphoton imaging. An Olympus x60 Plan-Apochromat oil immersion lens (Hachioji, Japan) with a numerical aperture of 1.4 was used for acquiring images. The 488 nm and 405 nm laser lines from a Kr/Ar laser were used to visualise the cells mounted on cover slipped slides. Emission was detected using a standard fluorescein filter (522 ± 35 nm) and a standard UV filter set (460 ± 10 nm) and a sequential scanning program. Images obtained by excitation with the 488 nm Kr/Ar laser were directed to the green channel, while images obtained by excitation with the 405 nm diode laser were directed to the blue channel. Channels were acquired sequentially, with a 4 s delay between scans and merged using Olympus Fluoview software (version 3).

## 4. Conclusions

Three NDI derivatives were studied in 1:1 (*v*/*v*) methanol/water as fluorescent pH indicators and DNA intercalators. The bay-substituted compounds are colourful and brightly fluorescent in acidic solution. In basic aqueous methanol, the compounds are weakly fluorescent with fluorescence quantum yields below naked-eye detection. Upon protonation, the tetra-substituted *N*,*N*-dimethylaminoethylene NDI is brightly fluorescent and functions as a dual-mode colorimetric and fluorescent pH indicator. Moreover, the tetra-substituted NDI causes a significant change in the CD ellipticity of CT-DNA. The strong DNA-binding ability of compound **3**, coupled with its efficient fluorescence response to DNA, identifies this cationic NDI derivative as a promising scaffold for the development of DNA-targeting fluorescent probes with potential theranostic agents. Future work will focus on the design of new NDI derivatives incorporating other lipophilic moieties to improve aqueous solubility while retaining their favourable photophysical properties and DNA-binding characteristics. The knowledge gained from this study opens the possibility of developing brighter and longer wavelength emitting naphthalenediimide fluorescent probes. Potential real-life applications are envisioned in cell imaging and oncology.

## Figures and Tables

**Table 1:** Optical data of compounds **1**–**3** in 1:1 (*v*/*v*) methanol/water by UV-visible absorption and steady-state fluorescence spectroscopy ^1^.

Parameter	1	2	3
λ_Abs_/nm (pH 11)	533	361, 382	594
log ε (mol^−1^ cm^−1^)	3.48	4.21	4.00
λ_Abs_/nm (pH 4)	533	360, 380	619 (598) [30]
log ε (mol^−1^ cm^−1^)	3.48	4.41	4.06 (4.11) [30]
λ_F_/nm (pH 4)	579	−	625
Δλ/nm ^2^	46	−	6
Apparent p*K*_a_ ^3^	−	−	6.0
*Φ*_Fmin_ ^4^	0.32	<0.001	0.002
*Φ*_Fmax_ ^4^	0.32	<0.001	0.38 (0.26) [30]
FE	1.0	−	190

^1^ Concentrations of 14.7 μM, 3.2 μM and 8.0 μM. ^2^ Stokes shift Δλ = λ_Flu_ − λ_Abs_ at pH 4. ^3^ Determined by $\left[\log_{10}\left(\frac{I_{\max}\;-\;I}{I\;-I_{\min}}\right)\right]=\;-\log\left[H^{+}\right]\;+\;\log\;\beta_{H^{+}}$ from emission spectra. ^4^ Relative *Φ*_Fmax_ with reference to the sodium salt of fluorescein in ethanol (0.79). Error ± 10%. Excitation wavelengths for compounds **1, 2** and **3** were 530 nm, 361 nm, and 594 nm, respectively.

## Data Availability

The original contributions presented in this study are included in the article/Supplementary Materials. Further inquiries can be directed to the corresponding author.

## Supplementary Materials

The following supporting information can be downloaded at https://www.mdpi.com/article/10.3390/molecules31183211/s1, Scheme S1: The synthesis of compounds **1**–**3**. Figure S1: ^1^H NMR spectrum of compound **1** in CDCl_3_ at 500 MHz, Figure S2: ^13^C NMR spectrum of compound **1** in CDCl_3_ at 126 MHz, Figure S3: ^1^H NMR spectrum of compound **2** in CDCl_3_ at 500 MHz, Figure S4: ^13^C NMR spectrum of compound **2** in CDCl_3_ at 126 MHz, Figure S5: ^1^H NMR spectrum of compound **3** in CDCl_3_ at 500 MHz, Figure S6: ^13^C NMR spectrum of compound **3** in CDCl_3_ at 126 MHz, Figure S7: FTIR spectrum of compound **1** as a thin film on a NaCl plate, Figure S8: FTIR spectrum of compound **2** as a thin film on a NaCl plate, Figure S9: FTIR spectrum of compound **3** as a thin film on a NaCl plate, Figure S10: Absorption (left) and emission (right) spectra excited at 537 nm on titration of 30.8 μM of compound **1** in 1:1 (*v*/*v*) methanol/water with increasing addition of CT-DNA (0 to 1.38 mM), Figure S11: Benesi–Hildebrant plot of emission data of 9.3 μM of compound **3** in aqueous methanol 1:1 (*v*/*v*) solution upon gradual addition of CT-DNA, Figure S12: Aqueous methanol 1:1 (*v*/*v*) solutions of 10^−5^ M of compounds **1**–**3** in a dark cabinet irradiated with 365 nm UV light.

## Abbreviations

The following abbreviations are used in this manuscript:

CD	Circular dichroism
CT	Calf-thymus
DNA	Deoxyribonucleic acid
ICT	Internal charge transfer
PET	Photoinduced electron transfer
NDI	Naphthalenediimide
