# Supplementary material for: Spectrophotometric Studies of a Fluorescent Naphthalenediimide PET and ICT pH Indicator and DNA Intercalator
Source: Molecules. 2026 Sep 11;31(18):3211. doi: 10.3390/molecules31183211 (PMC13609093; doi:10.3390/molecules31183211)
Supplement: Supplementary file 1 [file molecules-31-03211-s001.zip › molecules-4437339-supplementary.pdf]

Supplementary Materials

Molecules

Spectrophotometric Studies of a Fluorescent Naphthalenediimide PET and ICT pH Indicator and DNA Intercalator

Alex D. Johnson [1], Mario Valentino [2], Gary J. Hunter [2] and David C. Magri [1,*]

[1] Department of Chemistry, Faculty of Science, University of Malta, MSD 2080 Msida, Malta
[2] Department of Physiology and Biochemistry, Faculty of Medicine & Surgery, University of Malta, MSD 2080 Msida, Malta
* Correspondence: david.magri@um.edu.mt

Table of Contents

**1 Experimental**…………………………………………………………………...	2
**1.1 Materials**…………………………………………………………………………	2
**1.2 Instrumentation**……………………………………………………………....	2
**1.3 Cell Culturing** ……………………………………………….……….….....	3
**1.4 DNA Binding Studies**………………………………………………………….	4
**Scheme S1.** The synthesis of compounds **1-3**……………………………………………	5
**Figure S1**. ^{1}H NMR spectrum of compound **1** in $CDCl_3$ at 500 MHz……………..........	6
**Figure S2**. ^{13}C NMR spectrum of compound **1** in $CDCl_3$ at 126 MHz…………………	7
**Figure S3**. ^{1}H NMR spectrum of compound **2** in $CDCl_3$ at 500 MHz……………………	8
**Figure S4**. ^{13}C NMR spectrum of compound **2** in $CDCl_3$ at 126 MHz……………......	9
**Figure S5**. ^{1}H NMR spectrum of compound **3** in $CDCl_3$ at 500 MHz……………………	10
**Figure S6**. ^{13}C NMR spectrum of compound **3** in $CDCl_3$ at 126 MHz…………………	11
**Figure S7.** FTIR spectrum of compound **1** as a thin film on a NaCl plate…..……………	12
**Figure S8.** FTIR spectrum of compound **2** as a thin film on a NaCl plate…..……………	13
**Figure S9.** FTIR spectrum of compound **3** as a thin film on a NaCl plate…..……………	14
**Figure S10**. Absorption (left) and emission (right) spectra excited at 537 nm on titration of 30.8 μM of compound **1** in 1:1 (*v/v*) methanol/water with increasing addition of CT-DNA (0 to 1.38 mM)……………………………………………………………………	15
**Figure S11**. Benesi-Hildebrant plot of emission data of 9.3 μM of compound **3** in aqueous methanol 1:1 (v/v) solution upon gradual addition of CT-DNA………………	16
**Figure S12.** Aqueous methanol 1:1 (v/v) solutions of 10^{-5} M compounds **1-3** in a dark cabinet irradiated with 365 nm UV light…………………………………………………	17

1 Experimental

1.1 Materials

RPMI 1640 Medium was obtained as a powder from Gibco BRL, Life Technologies Ltd. UK. RPMI 1640 Medium is ready mixed in sealed aliquots to make up 1 L of medium containing supplemented with 2 mM *L*-glutamine, 1000 mg/L *d*-glucose, 110 mg/L sodium pyruvate, 25 mM HEPES, without sodium hydrogen carbonate. RPMI 1640 supplemented with 2 mM L-glutamine, 1 mM sodium pyruvate, without sodium hydrogen carbonate.

Penicillin-Streptomycin were obtained from Gibco BRL, Life Technologies Ltd., UK. The Penicillin-Streptomycin lyophilised preparation was rehydrated with sterile distilled water and stored at -20 °C. The preparation provides 10,000 units/mL of Penicillin G (sodium salt) and 10,000 µg/mL of Streptomycin Sulphate. Gibco recommends use of 50-100 units /mL of sterilized medium or 1-2 mL of the rehydrated solution with every 200 mL of sterilized medium.

The Foetal Bovine Serum (FBS) used as a serum-supplement for *in vitro* cell culture was obtained from Gibco BRL, Life Technologies Ltd., UK. It was heat treated, virus, and mycoplasma screened before use and stored at -20°C. and added to 200 mL medium aliquots. Other reagents included sodium hydrogen carbonate and sterile 1 N hydrochloric acid and 1 N sodium hydroxide. The cell culture MCF-7 was obtained from the European Collection of Authenticated Cell Culture (ECACC).

1.2 Instrumentation

Reactions were carried out in single, two-neck, and three necked round bottom flasks (50 mL, 100 mL or 250 mL) partially immersed in mineral oil and heated using an IKA C-MAG HS 7 hotplate fitted with an IKA ETS-D5 temperature probe. Melting points of solids were recorded using a Stuart SMP40 automatic melting point apparatus calibrated against pure samples of vanillin and phenacetin. Melting point measurements were taken using the rapid melt mode and accurate measurements with a ramp rate of 0.1 °C/min with the melt videos automatically saved as an AVI file for viewing on the unit or via PC.

^{1}H and ^{13}C NMR spectra were recorded on a Bruker Avance III HD NMR spectrometer equipped with an Ascend 500 11.75 Tesla superconducting magnet operating at a frequency of 500.13 MHz for ^{1}H NMR and 125.76 MHz for ^{13}C NMR with a multinuclear 5

mm PABBO Probe. Data were acquired and processed via Topspin Software, ver. 3.2. Chemical shifts for ^{1}H NMR were recorded in ppm downfield from TMS set at $\delta = 0.00$ ppm and ^{13}C NMR spectra were referenced to the solvent triplet signal of $CDCl_3$ at $\delta = 77.00$ ppm.

UV-visible absorption spectra were recorded on a Jasco V-650 spectrophotometer connected to a desktop computer. Samples were measured in 10 mm Suprasil quartz cuvettes. The instrumentation parameters were set to medium response, bandwidth of 2 nm and scan speed of 200 nm min^{-1}. All spectra were background corrected for the solvent by scanning the appropriate blank solvent prior to beginning the experiments. Fluorimetric studies were conducted using a Jasco FP-8300 spectrofluorometer with a 10 mm path length Suprasil quartz cuvette. The excitation and emission slits were set between 2.5 nm and 5.0 nm. The scan rate was 200 nm min^{-1}.

1.3 Cell Culturing

Sterile conditions for cell cultures were maintained by a Laminar Flow Hood (Microflow Advanced) with the provided High Efficiency Particulate Air (HEPA), and filtered sterile air circulate within an enclosed space. Instrumentation required for cell culture sterilisation, and preparation of medium included a Millipore™ Pressure Vessel for positive-pressure filtration system, Milli-Q^{eu}™ Deionized unit, pH meter with temperature probe, magnetic stirrer and plastic-coated stirrer bars, and electronic balance. The following apparatus were used to sterilise the cell medium; Pipette filler Pump Operator (Automatic), filter units with filling bell, 0.22 μm Sterivex ™ - GS (Sterile), and filter units, syringe adapted, 0.2 μm cellulose acetate membrane. Disposable syringes of 5 mL, 10 mL, and 20 mL, along with 1 L measuring jar and beaker, 1 L Erlenmeyer flask, and 250 mL medium bottles (sterile).

An ELx – 808, Bio Tek microplate reader was used to shake and read the optical density at 650 nm of the 96-well plates after MTT assays. An Axiovert 40 CLF microscope, manufactured by Carl Zeiss MicroImaging was used for phase contrast viewing to obtain cell counts. The ocular magnification is ×10, whilst the objective magnifications are ×4, ×10, ×40 and ×60. The cell imaging interface software for the microscope is AxioVision Rel. version 4.8.1 (11-2009). Rotofix 32A was required for the cytospin of suspension cells (K562) on glass slides for morphology fluorescent staining experiments. Fluorescent imaging was captured using an EVOS FL Auto Cell Imaging system fluorescent microscope.

1.4 DNA Binding Studies

The binding constants with CT-DNA to form a complex were determined from the Benesi-Hildebrand equation:

$$\frac{1}{A_0 - A} = \frac{1}{K(A_0 - A_F)} \times \frac{1}{[DNA]} + \frac{1}{A_0 - A_F}$$

where K is the binding constant, A_0 is the initial absorbance in the absence of DNA, A is the observed absorbance on addition of DNA and A_F is the final absorbance on addition of DNA at saturation. A linear plot of $1/(A_0 - A)$ versus 1/[DNA] was constructed from the titration data and fitted to a linear regression analysis. From the slope, $m = 1/K(A_0 - A_F)$ the binding constant was calculated. A similar analysis was performed using the fluorescence intensities rather than absorbances.

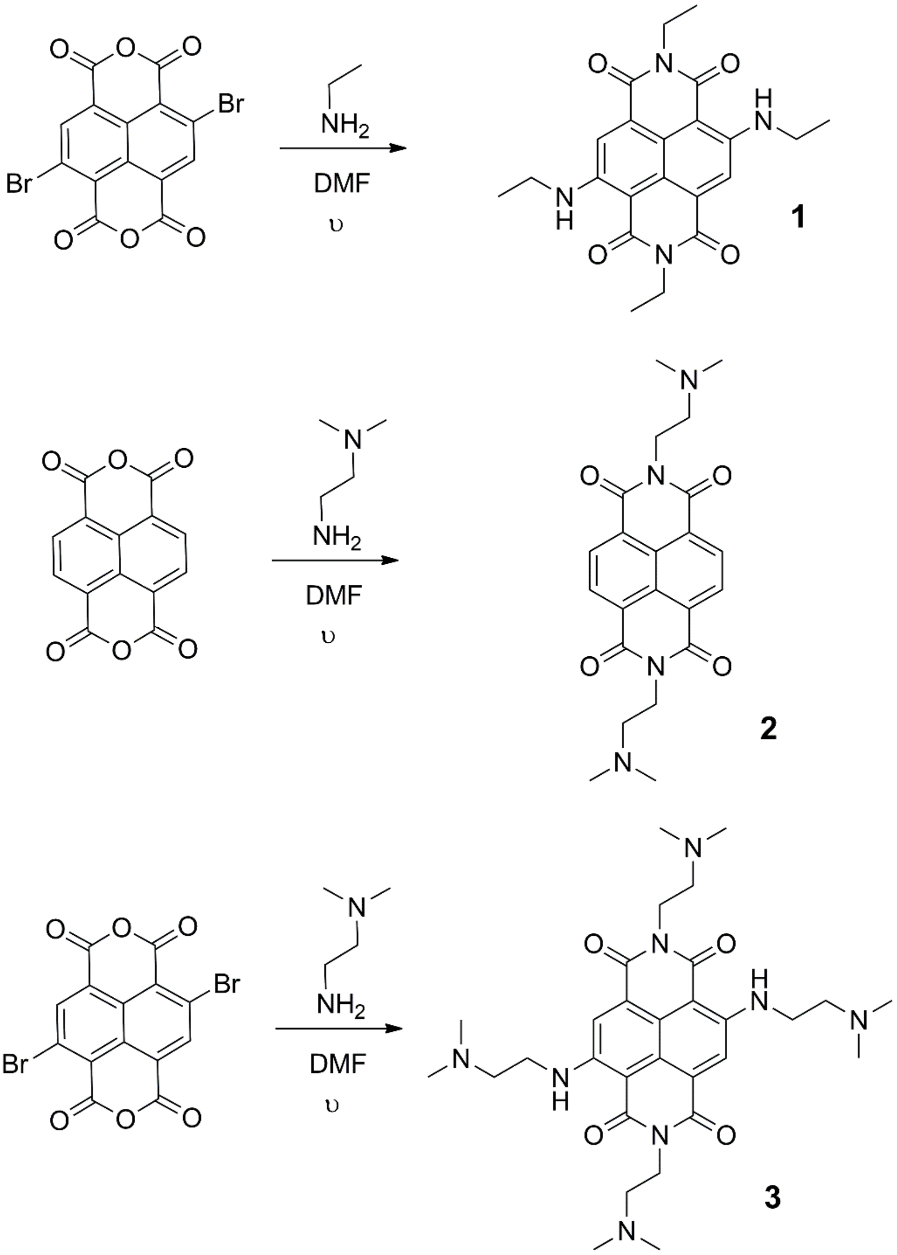

Scheme S1. The synthesis of compounds **1-3.**

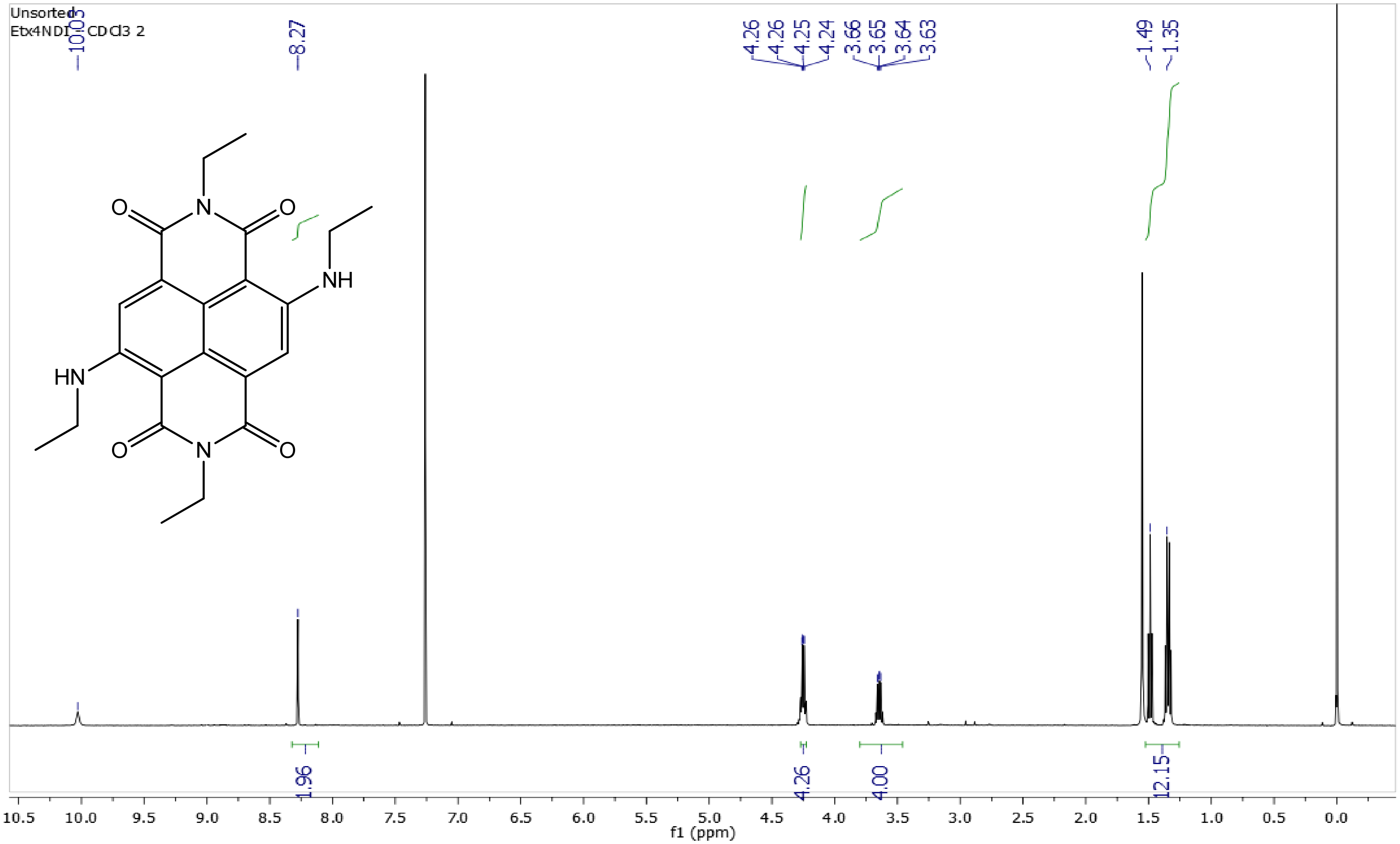

Figure S1. ^{1}H NMR spectrum of compound **1** in $CDCl_3$ at 500 MHz.

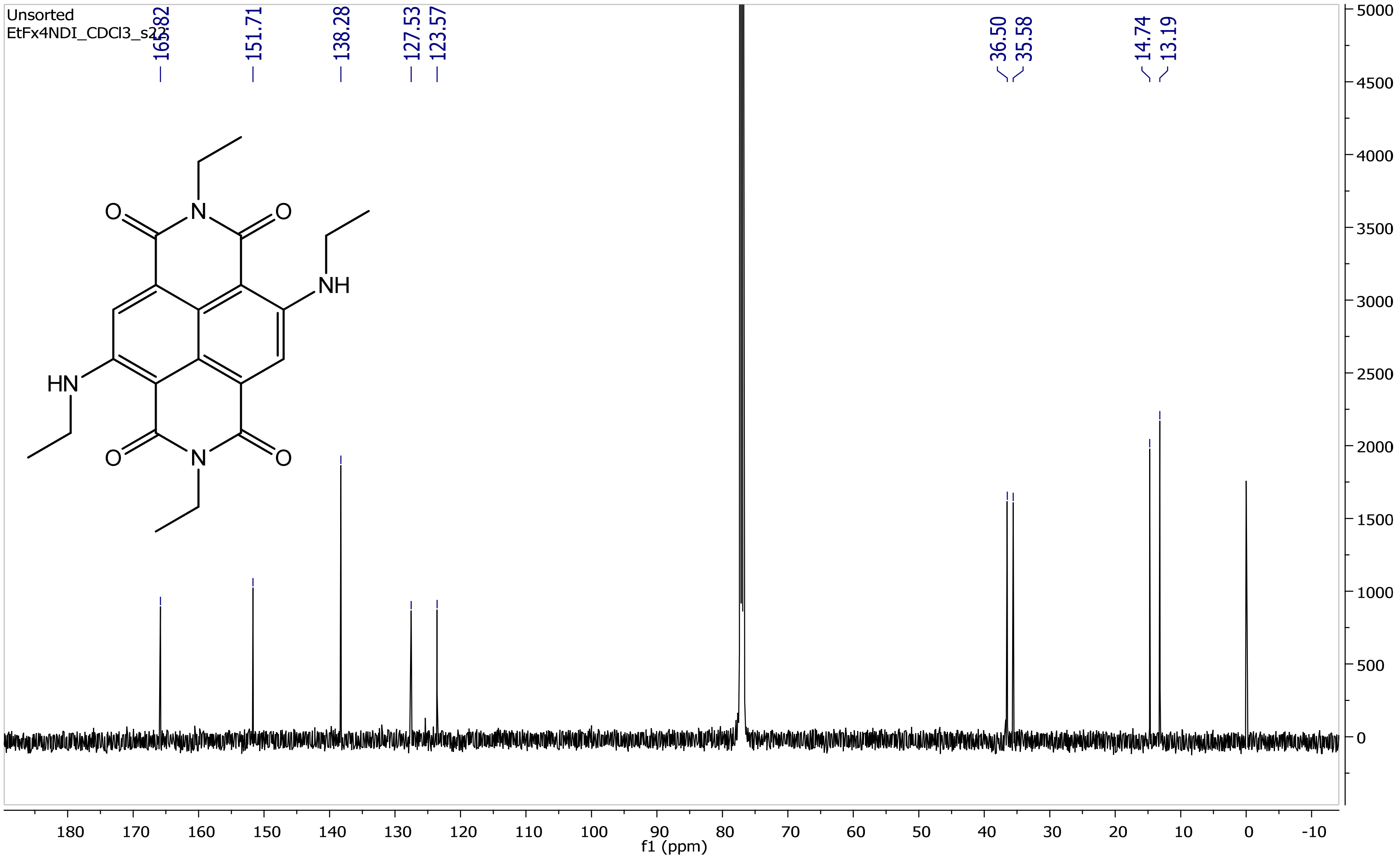

Figure S2. ^{13}C NMR spectrum of compound **1** in $CDCl_3$ at 126 MHz.

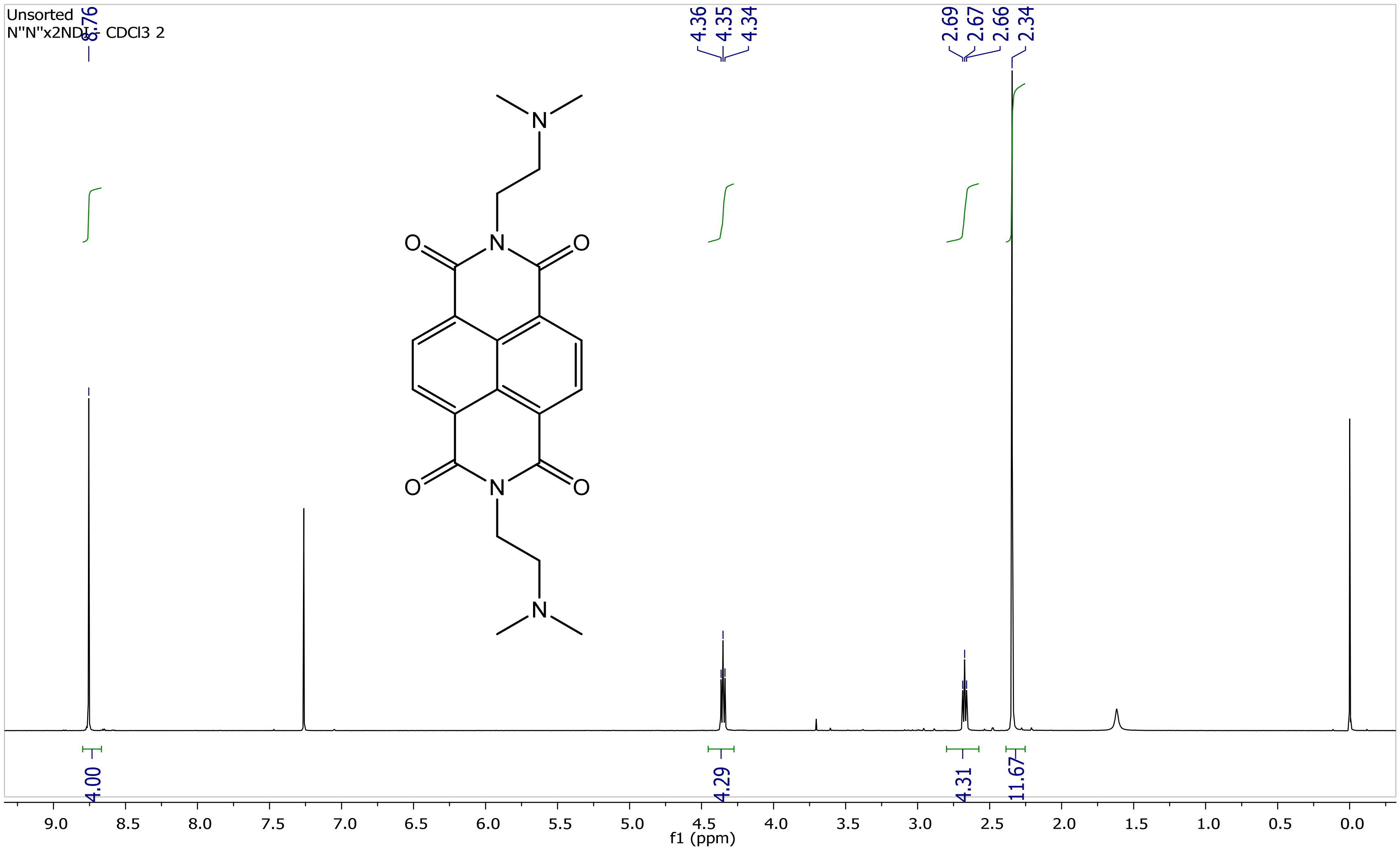

Figure S3. ^{1}H NMR spectrum of compound **2** in $CDCl_3$ at 500 MHz.

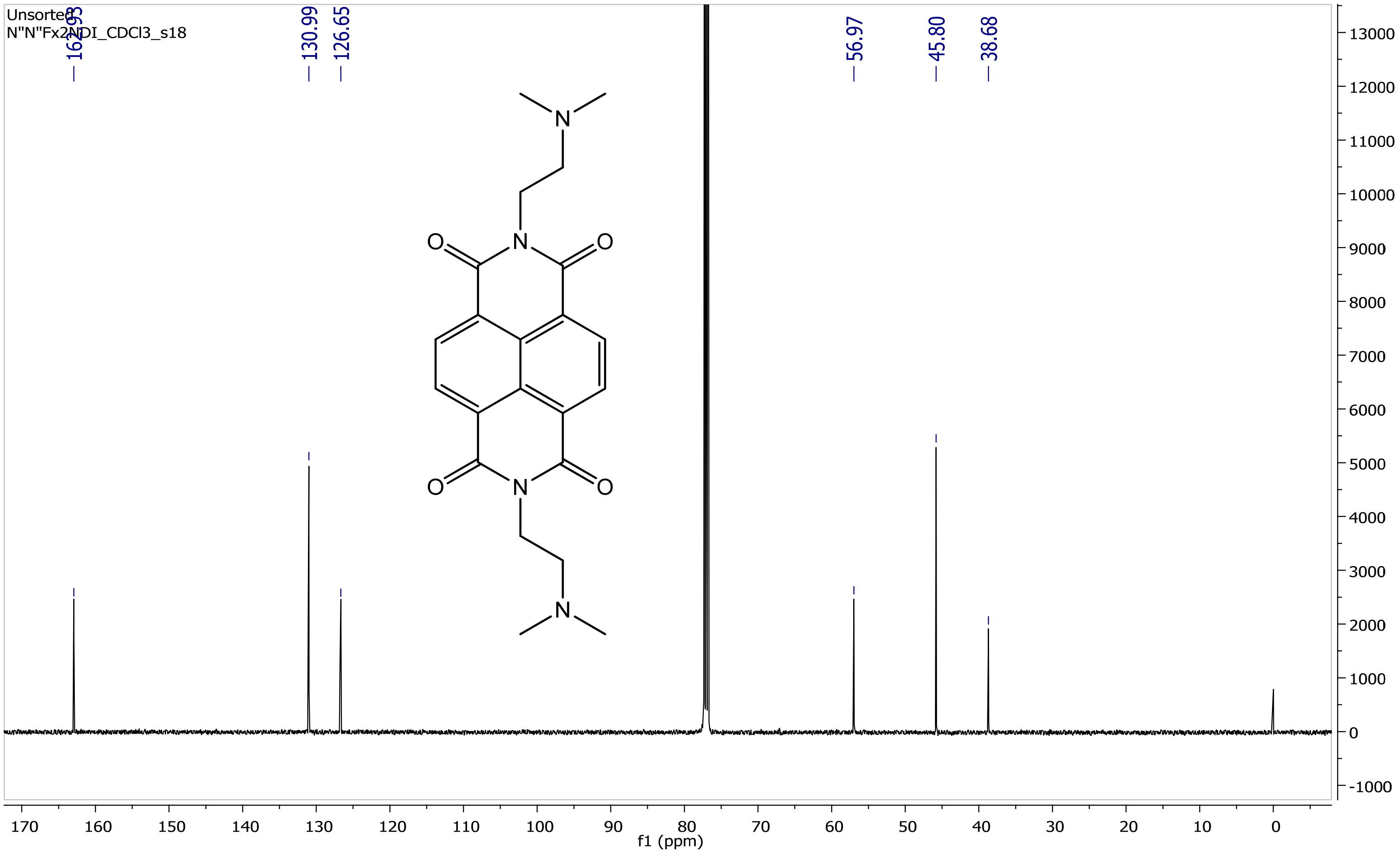

Figure S4. ^{13}C NMR spectrum of compound **2** in $CDCl_3$ at 126 MHz.

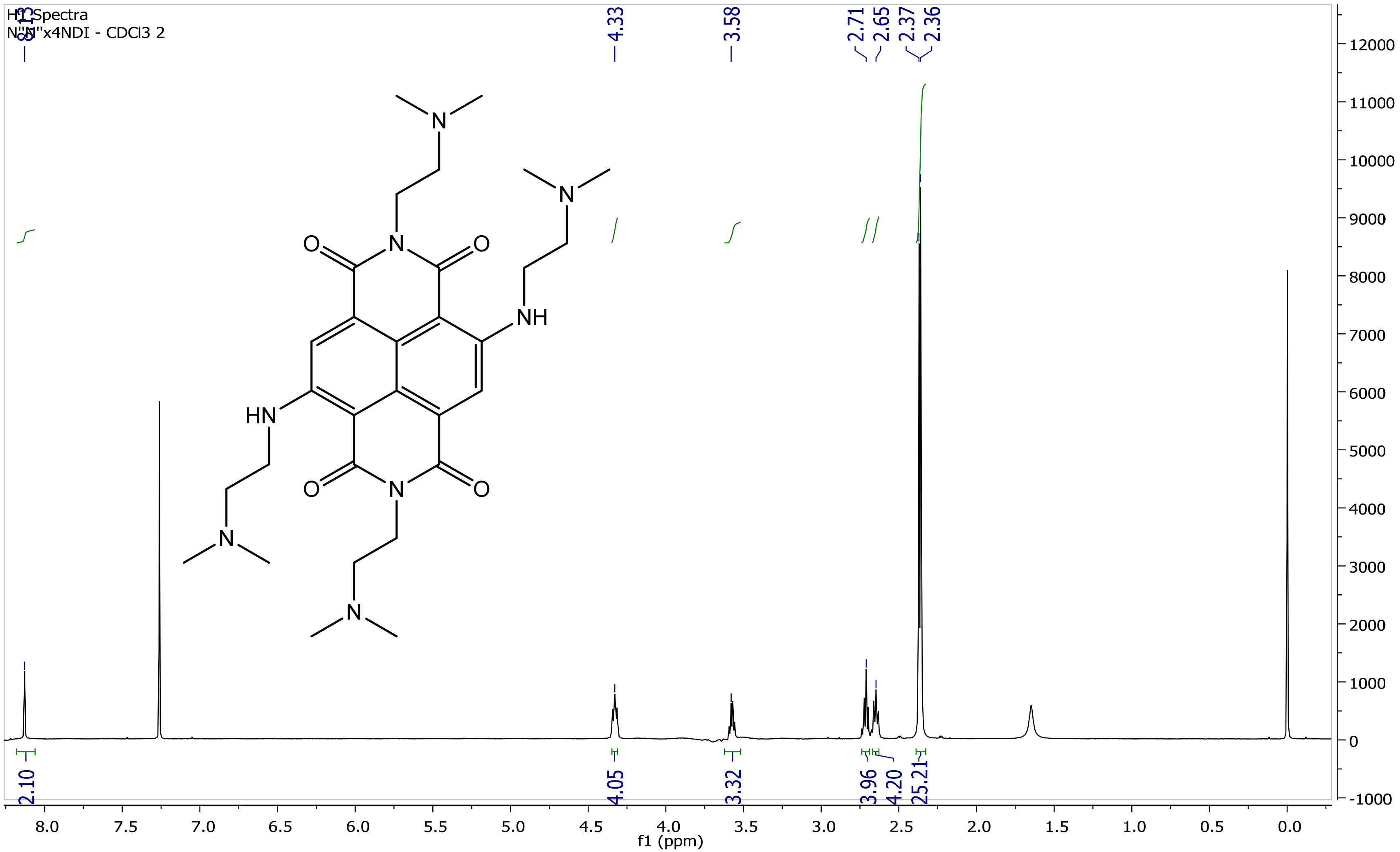

Figure S5. ^{1}H NMR spectrum of compound **3** in $CDCl_3$ at 500 MHz.

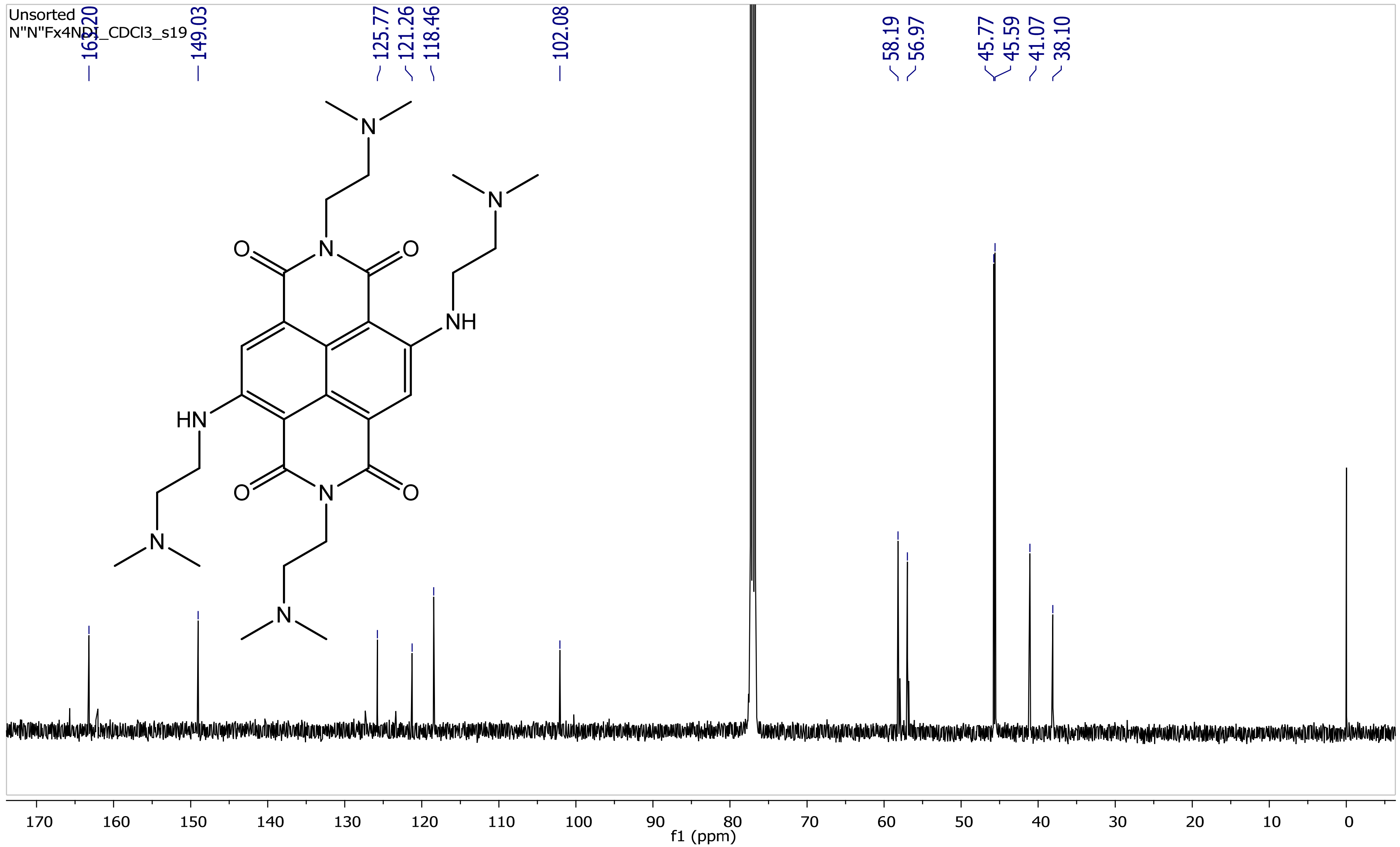

Figure S6. ^{13}C NMR spectrum of compound **3** in $CDCl_3$ at 126 MHz.

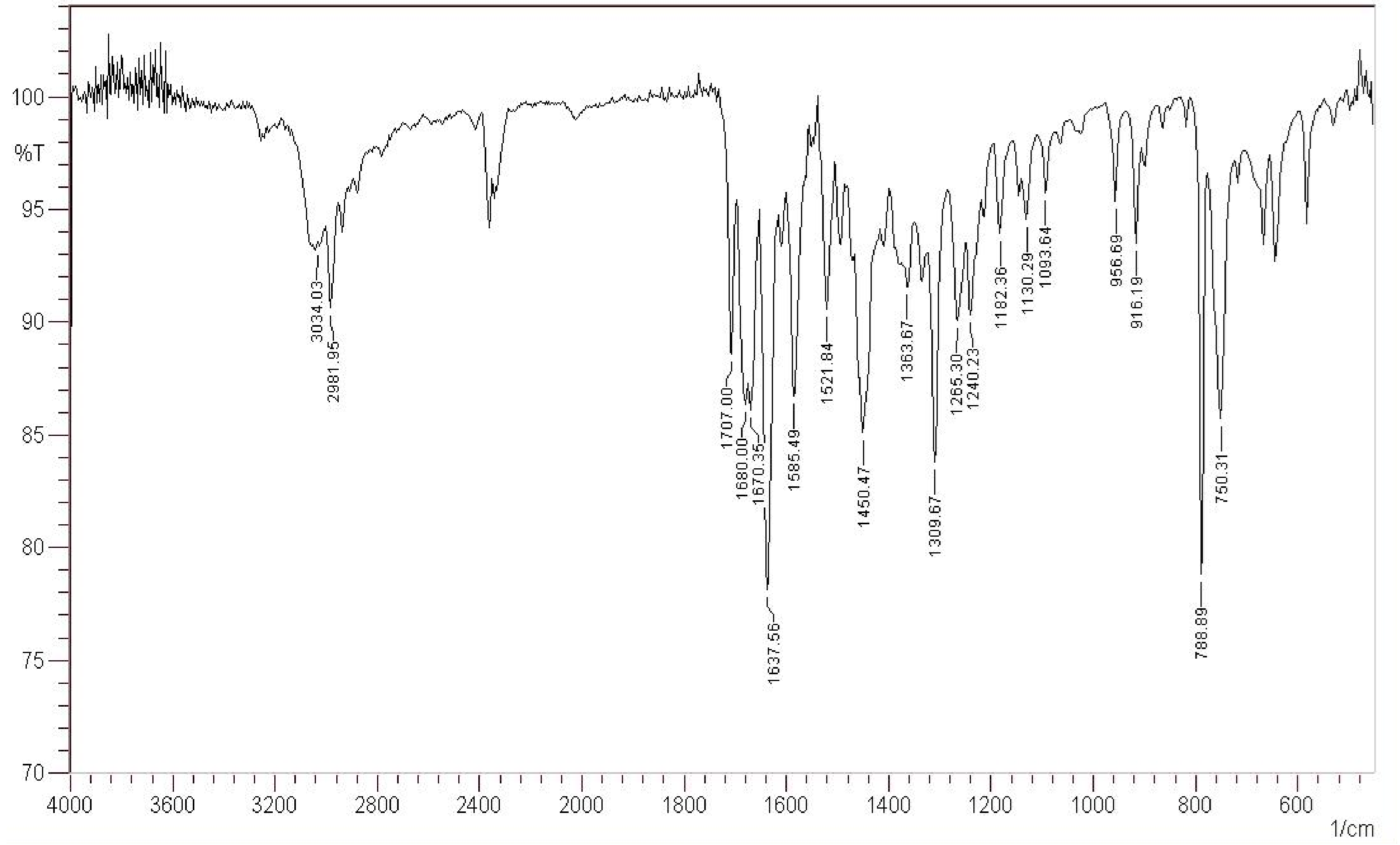

Figure S7. FTIR spectrum of compound **1** as a thin film on a NaCl plate.

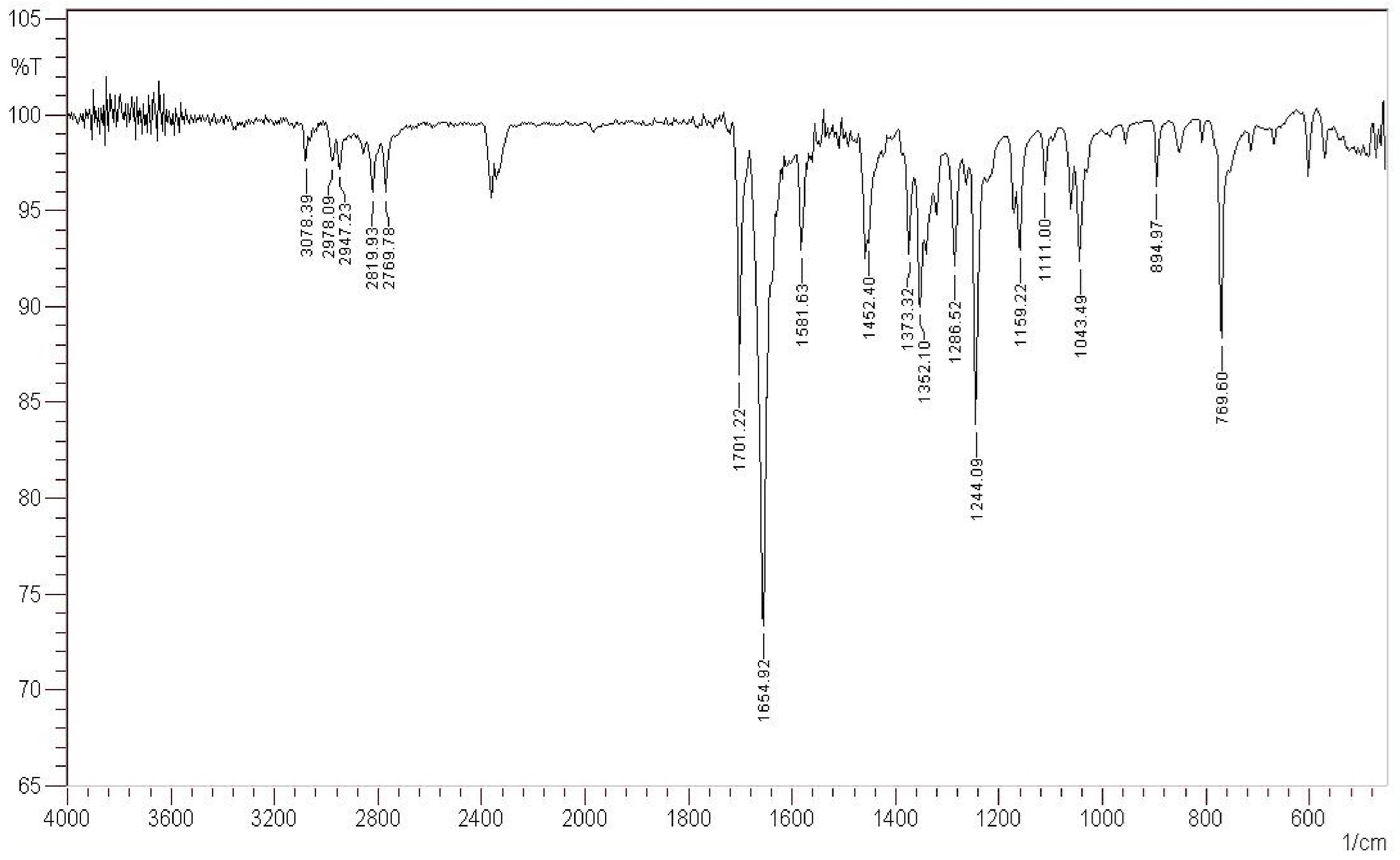

Figure S8. FTIR spectrum of compound **2** as a thin film on a NaCl plate.

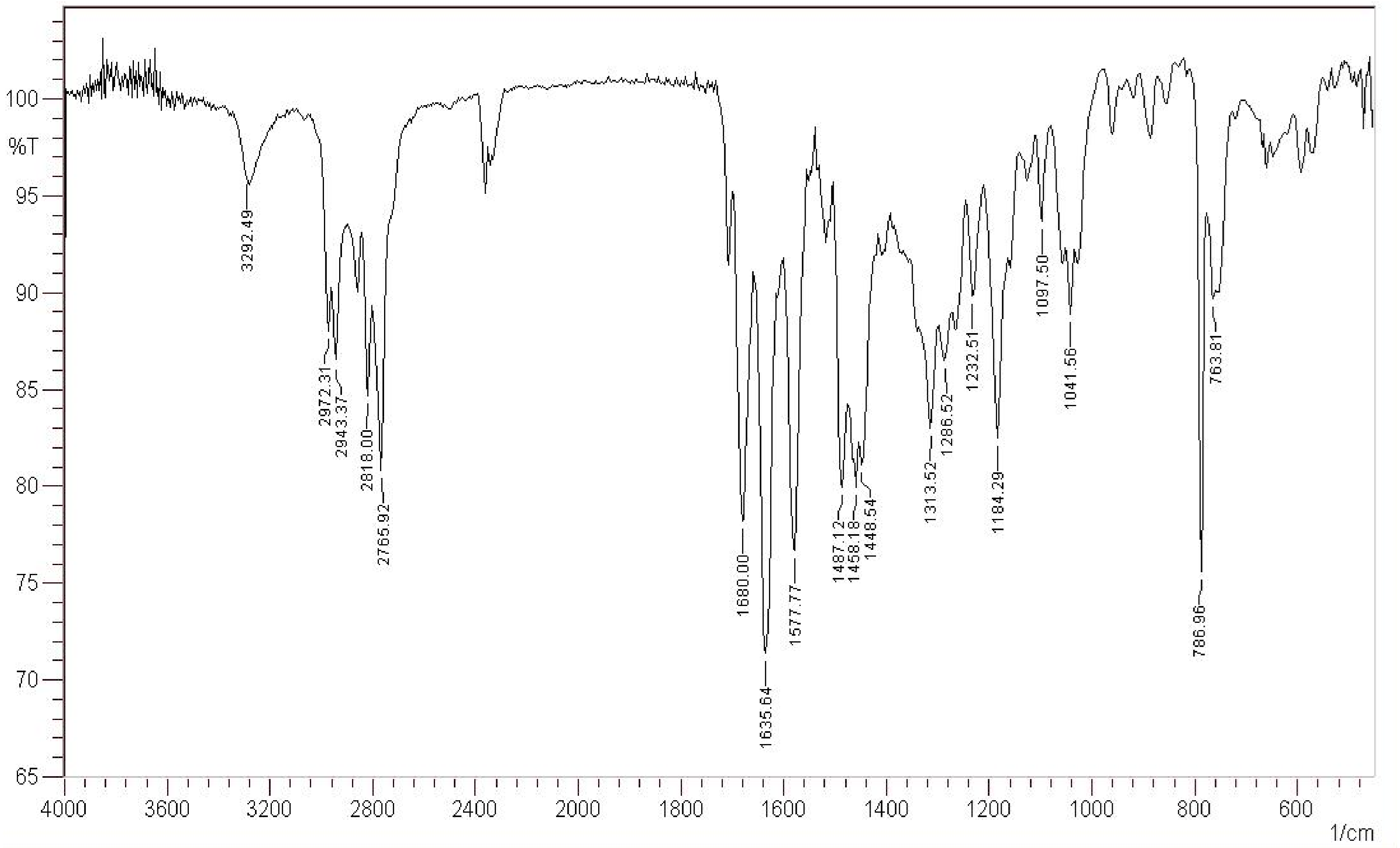

Figure S9. FTIR spectrum of compound **3** as a thin film on a NaCl plate.

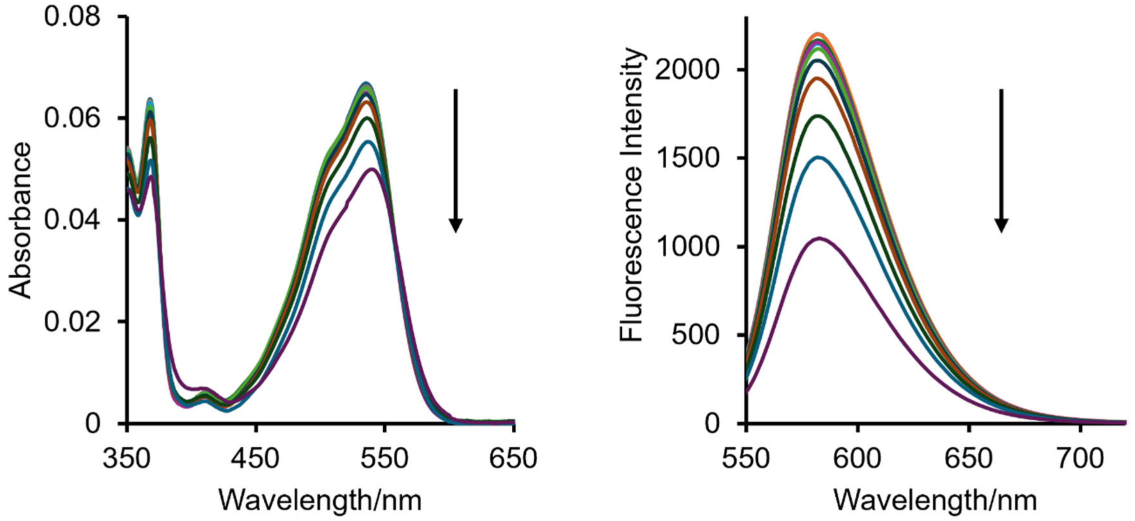

Figure S10 Absorption (left) and emission (right) spectra excited at 537 nm on titration of 30.8 μM of compound **1** in 1:1 (*v/v*) methanol/water with increasing addition of CT-DNA (0 to 1.38 mM).

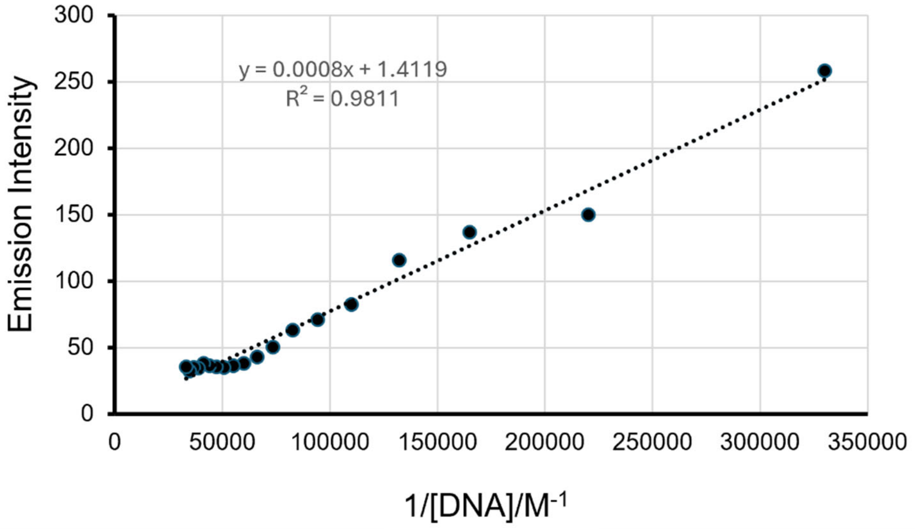

Figure S11 Benesi-Hildebrant plot of emission data of 9.3 μM of compound **3** in aqueous methanol 1:1 (v/v) solution upon gradual addition of CT-DNA.

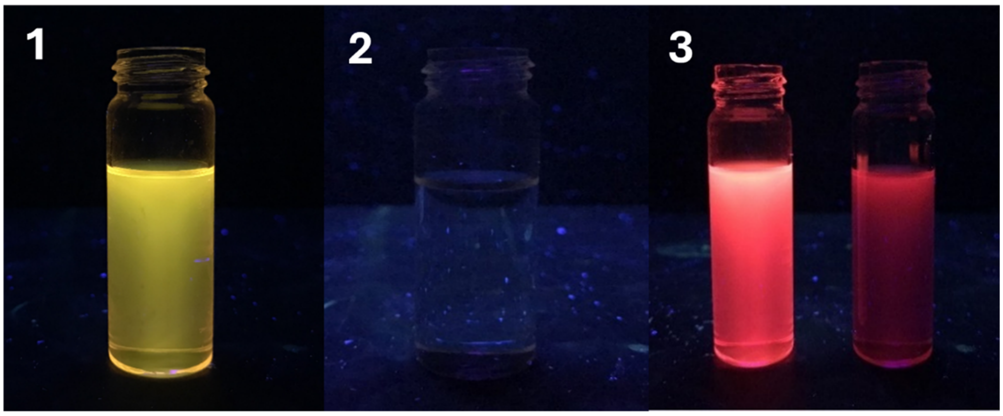

Figure S12 Aqueous methanol 1:1 (v/v) solutions of 10^{-5} M compounds **1-3** in a dark cabinet irradiated with 365 nm UV light.
